# Deficiency of placental GDF15 causes fetal growth restriction by inhibiting fetal hepatic STAT3-IGF2 axis

**DOI:** 10.1371/journal.pbio.3004061

**Published:** 2026-10-14

**Authors:** Xiao Peng Liu, Xiao Zhang, Chun Xia Cai, Miao Miao Qin, Xiang Kai Leng, Jia Hao Chen, Yi Fan Han, Jun Ying Xiao, Jia He Huang, Jiang Wei Wu

**Affiliations:** Key Laboratory of Animal Genetics, Breeding and Reproduction of Shaanxi Province, College of Animal Science and Technology, Northwest A&F University, Yangling, Shaanxi, China; INSERM, FRANCE

## Abstract

Fetal growth restriction (FGR) is a major cause of perinatal morbidity and mortality, yet its underlying mechanism remains elusive. Here, we show that growth differentiation factor 15 (GDF15) levels are significantly reduced in the placenta and fetal circulation in mouse and pig FGR models and correlate positively with fetal weight. Placenta-specific GDF15 deficiency causes FGR, while placenta-specific *Gdf15* mRNA delivery through targeted lipid nanoparticle (LNP) effectively alleviates FGR. Mechanistically, placental GDF15 is a major contributor to the fetal circulating GDF15 pool and enters the fetal compartment, where it activates the hepatic STAT3 signaling pathway to stimulate IGF2 production during pregnancy. Placental GDF15 deficiency decreases hepatic STAT3 phosphorylation and IGF2 levels in fetuses. Inhibition of STAT3 in primary mouse fetal hepatocytes blocks GDF15-induced IGF2 upregulation. Placenta-specific *Gdf15* mRNA-LNP administration rescues the decreased hepatic p-STAT3 and IGF2 levels in FGR fetuses. Collectively, our findings identify a placenta-fetal liver metabolic axis where placental GDF15 promotes fetal growth by activating the fetal hepatic STAT3-IGF2 pathway, revealing a potential therapeutic target for FGR.

## Abstract 2

Circulating levels of GDF15 change during pregnancy, and low levels have been linked to fetal growth restriction in clinical studies. This study shows that placenta-specific GDF15 is required to support fetal growth in mice, and that it enters the fetal compartment to regulate hepatic metabolism.

## Introduction

Fetal growth restriction (FGR) is a common pregnancy complication, affecting ~10% of pregnancies worldwide and characterized by impaired fetal growth and low birth weight [1,2]. It predisposes offspring to diseases such as cardiovascular disease [3], diabetes [4] and cognitive disorder [5,6]. Fetal growth is regulated by the coordinated interplay of maternal, placental, and fetal factors [7]. Among these, the placenta serves as an essential interface that provides nutrient support and endocrine regulation for fetal growth [8,9]. Growth differentiation factor 15 (GDF15), also known as placental bone morphogenetic protein [10], is implicated in diverse diseases including obesity [11–13], cancer cachexia [14,15], and kidney injury [16]. In recent years, its role in gestation has garnered significant attention. During pregnancy, plasma GDF15 is mainly originated from the feto-placental unit [17], specifically the trophoblasts [18–20]. Circulating GDF15 levels are markedly elevated in the mid-to-late gestation in mammals [19–21], implying a potential role for GDF15 in regulating fetal growth. In pathological state, human and animal studies have demonstrated that maternal circulating or placental GDF15 levels strongly link to the risk of early reproductive disorders such as miscarriage [22,23] and hyperemesis gravidarum [17]. Exogenous supplementation increases in GDF15 alleviated pregnancy failure in mice [24], highlighting its critical role in maintaining early pregnancy. Notably, a recent clinical study has showed markedly reduced plasma GDF15 levels in late gestation in cohorts of small-for-gestational-age patients whose babies with low birthweight [25], indicating that GDF15 may be associated with FGR. However, whether and how placenta-derived GDF15 modulates FGR remains unclear.

Fetal growth is centrally regulated by the growth hormone (GH)/insulin-like growth factor (IGF) axis [26,27]. IGF family members, particularly IGF1 and IGF2, are synthesized primarily by the liver [28]. They regulate cell proliferation and differentiation by activating the IGF-1 receptor (IGF-1R) to drive fetal growth [29,30]. Multiple evidence from humans have demonstrated that reduced IGF1 and IGF2 levels in fetal circulation are strongly linked to FGR [31,32]. Moreover, targeted delivery of IGF2 to the placenta of pregnant mice using liposomes effectively ameliorated FGR [33]. The relationship between GDF15 and GH signaling has been shown in one previous study, in which cardiomyocytes-derived GDF15 acts as an endocrine signal to inhibit hepatic GH signaling and body growth in pediatric heart disease [34]. Meanwhile, a study on blood protein analysis of healthy women with term pregnancy found the presence of GDF15 in umbilical vein blood [35], implying that GDF15 derived from the placenta might enter the fetal circulation to exert an endocrine effect. Based on these findings, it is highly plausible that during pregnancy, placenta-derived GDF15 may act as an endocrine signal to modulate the activity of the GH/IGF axis in the fetuses, thereby affecting fetal growth.

In this study, we combined low-protein diet-induced FGR mice, naturally occurring porcine FGR piglets, and *Gdf15* gene-edited mice to investigate the role of placental GDF15 in FGR. We found that placental and fetal circulating GDF15 levels were significantly reduced in these FGR models. Systemic or placenta-specific GDF15 deficiency resulted in FGR, whereas placenta-specific *Gdf15* overexpression effectively alleviated the FGR phenotype. Mechanistically, placental GDF15 was transferred into the fetal circulation and preferentially accumulated in the fetal liver. Conducting fetal liver proteomic analysis and experimental validation, we identified fetal hepatic IGF2 as a key downstream mediator of placental GDF15. Pathway enrichment analysis and validation revealed that placental GDF15 deficiency inhibits the hepatic STAT3 signaling pathway. Inhibition of STAT3 signaling in primary mouse fetal hepatocytes abolished GDF15-induced IGF2 upregulation. Placenta-specific *Gdf15* mRNA-LNP administration to FGR pregnant mice rescued the reduced p-STAT3 and IGF2 protein levels in the fetal liver. Altogether, these findings reveal the crucial role of placental GDF15-fetal hepatic STAT3-IGF2 endocrine axis in driving fetal growth and highlight it as a promising therapeutic target for FGR.

## Results

### Placental and fetal circulating GDF15 levels are reduced in mouse and pig FGR models

GDF15 is highly expressed in the placentas [18,36] during pregnancy. To investigate whether placental GDF15 is associated with FGR, we constructed two types of FGR mouse models [6] induced by protein restriction diet and Dexamethasone, respectively. Successful induction of FGR was confirmed by reduced fetal weight, short crown-rump length, decreased fetal liver weight, placental weight and placental efficiency at embryonic 18.5 days (E18.5) (Figs 1A and S1A–S1J), although no significant differences in the embryo number and resorption rate compared to controls (S1K and S1L Fig). Placental GDF15 expression and fetal plasma GDF15 levels were significantly decreased in FGR mice compared to normal controls (Figs 1B–1E, S1M, and S1N), and were positively correlated with fetal weight (Fig 1F and 1G). Maternal plasma GDF15 levels were also markedly decreased in FGR pregnant mice (S1O and S1P Fig). Consistent with these findings in mice, placental GDF15 expression and circulating GDF15 levels in FGR piglets were also significantly downregulated (Fig 1H–1L), and were positively correlated with birth weight (Fig 1M and 1N). Together, these results emphasize the potential role of GDF15 in FGR.

**Fig 1:**
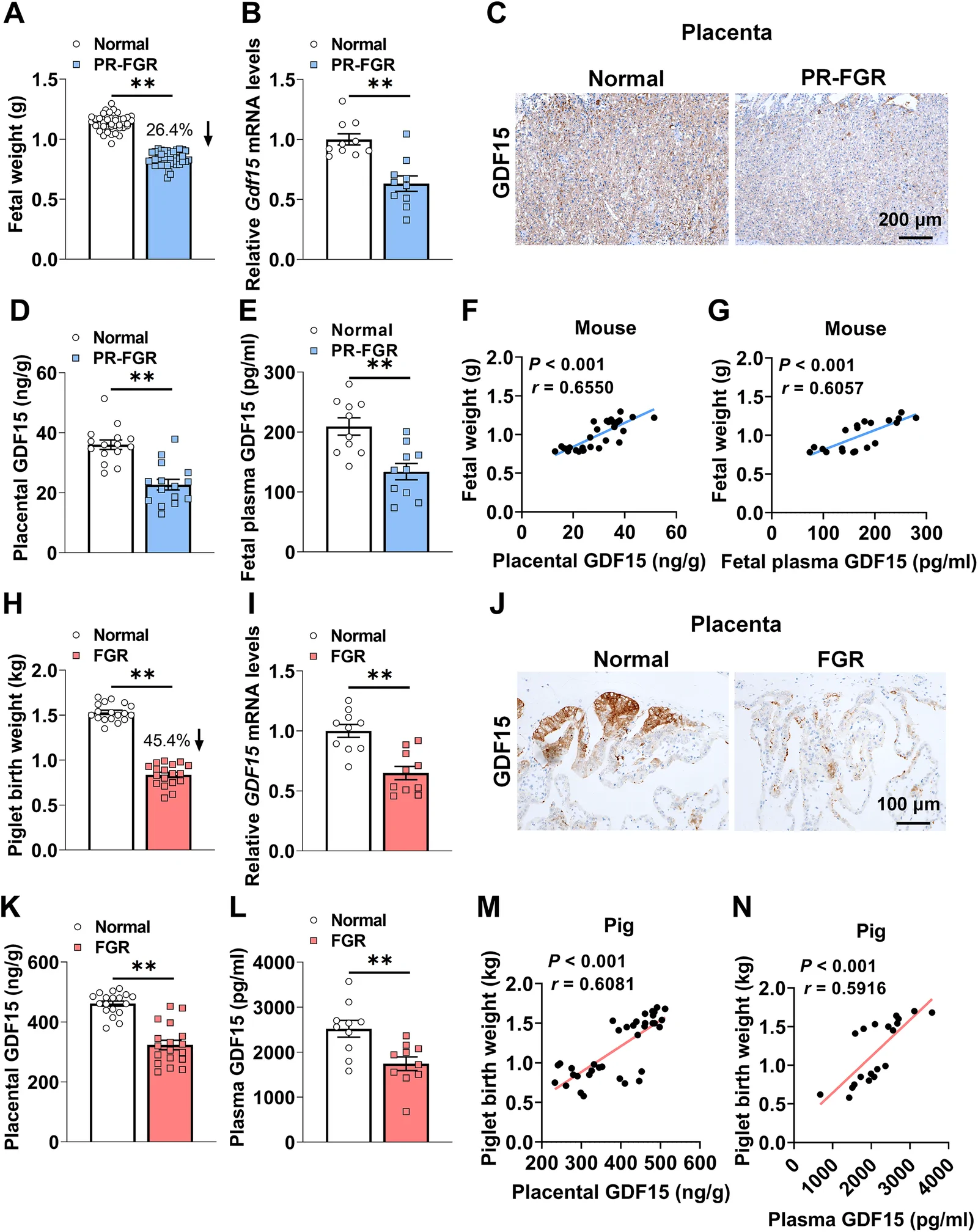
Placental and fetal plasma GDF15 levels are reduced in FGR models and correlate with fetal weight across different species. **(A)** Fetal weight in normal (*n* = 42) and PR-FGR (*n* = 39) mice. FGR mice model was induced by protein restriction diet (8%). **(B)** qPCR analysis to quantify placental *Gdf15* mRNA expression from normal and PR-FGR groups (*n* = 10). **(C)** Representative immunohistochemical staining for GDF15 in placental sections of fetal mice in normal and PR-FGR groups. Scale bar, 200 μm. **(D)** ELISA analysis to quantify placental GDF15 levels in each group (*n* = 15 from 5 litters). **(E)** ELISA analysis to quantify plasma GDF15 levels of fetal mice in each group (*n* = 10 from 5 litters). **(F)** Pearson’s correlation coefficient analysis of placental GDF15 levels and body weight in fetal mice at E18.5 (*n* = 30). **(G)** Pearson’s correlation coefficient analysis of plasma GDF15 levels and body weight in fetal mice at E18.5 (*n* = 20). **(H)** Body weight of newborn piglets in normal and FGR groups (*n* = 18). **(I)** qPCR analysis to quantify placental *GDF15* mRNA expression from normal and FGR groups (*n* = 10). **(J)** Representative immunohistochemical staining for GDF15 in placental sections of newborn piglets in normal and FGR groups. Scale bar, 100 μm. **(K)** ELISA analysis to quantify placental GDF15 levels of piglets in each group (*n* = 18). **(L)** ELISA analysis to quantify plasma GDF15 levels of newborn piglets in each group (*n* = 10). **(M)** Pearson’s correlation coefficient analysis of placental GDF15 levels and body weight in newborn piglets (*n* = 36). **(N)** Pearson’s correlation coefficient analysis of plasma GDF15 levels and body weight in newborn piglets (*n* = 20). Data are presented as the mean ± SEM. *P* values were calculated by two-tailed unpaired Student *t* test (A, B, D, E, H, I, K, L) or Pearson correlation (F, G, M, **N)**. *P < 0.05, **P < 0.01. PR-FGR, protein restriction diet-induced FGR; E18.5, embryonic 18.5 days. The underlying data for this figure can be found in S1 Data.

### Placental GDF15 deficiency impairs fetal growth in utero

To determine the role of GDF15 in FGR, global *Gdf15*-knockout mice were generated and verified by PCR genotyping (S2A and S2B Fig). We first employed reciprocal crossing schemes in which wild-type (WT) (*Gdf15*^+/+^) female mice × homozygous (*Gdf15*^*−/−*^) male mice and the *Gdf15*^−/−^ female mice × WT male mice to investigate whether maternal GDF15 regulates fetal growth (S2C Fig). We found that plasma GDF15 levels in heterozygous fetuses from WT dams were comparable to those from *Gdf15*-null dams (S2D Fig), indicating that maternal GDF15 deficiency does not substantially reduce fetal circulating GDF15. Maternal GDF15 deficiency did not significantly alter fetal weight and crown-rump length at E18.5 (S2E–S2G Fig). Moreover, there is no differences in the embryo number and resorption rate between the two groups (S2H and S2I Fig). These data indicate that maternal-derived GDF15 may be not required to maintain fetal growth or fetal circulating GDF15 levels.

To further investigate whether the role of fetal-placental unit-derived GDF15 in FGR, we analyzed the growth phenotypes of fetuses with different *Gdf15* genotypes (*Gdf15*^+/+^, *Gdf15*^+/−^, *Gdf15*^−/−^) at E18.5, which had been produced by using a heterozygous × heterozygous breeding scheme (Fig 2A). Placental and fetal plasma GDF15 levels, along with the growth parameters including body weight and crown-rump length in *Gdf15*^−/−^ fetuses were significantly lower than those in wild-type (*Gdf15*^+/+^) or heterozygous (*Gdf15*^+/−^) fetuses (Fig 2B–2G). No significant differences in the placental weight among them (Fig 2H), whereas GDF15 deficiency significantly decreased placental efficiency (Fig 2I). In addition, there is no significant differences in fetal growth parameters and placental parameters were shown between *Gdf15*^+/+^ and *Gdf15*^+/−^ fetuses (Fig 2F–2I). These data suggest that GDF15 deficiency impairs fetal growth. We further assessed the postnatal growth effect of GDF15 deficiency. *Gdf15*-deficient pups exhibited low birth weight compared to *Gdf15*^+/+^ or *Gdf15*^+/−^ (Fig 2J), and the differences were also observed at weaning (Fig 2K). These findings demonstrate that GDF15 derived from fetal-placental unit increases fetal circulating GDF15 levels and regulates fetal and early postnatal growth.

**Fig 2:**
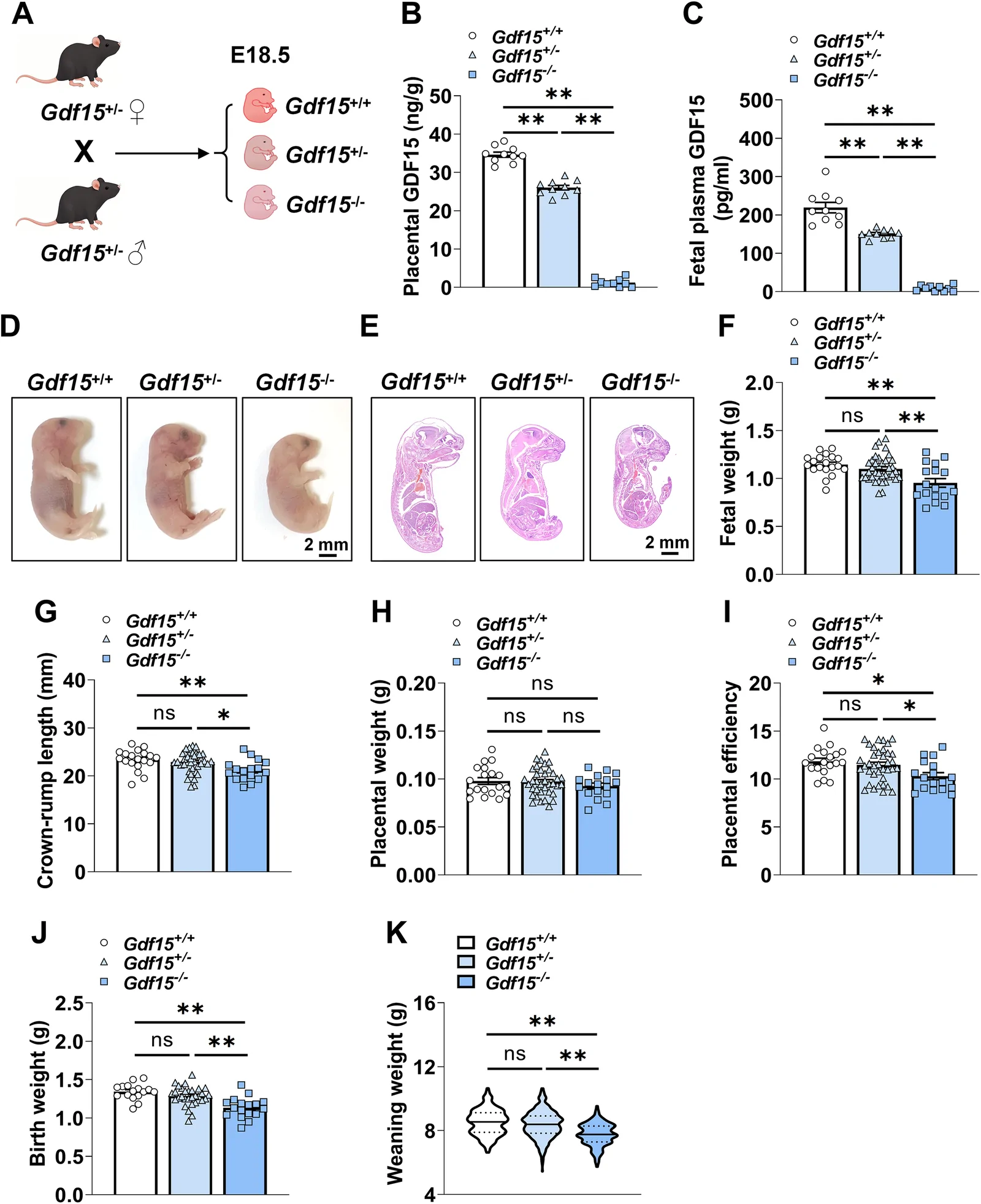
GDF15 deficiency causes defective fetal growth phenotype in pregnant mice. **(A)** Schematic diagram of breeding strategy. Generating offspring mice of different *Gdf15*-genotypes using 8–10 weeks male and female *Gdf15*-heterozygous mice (*N* = dams, *n* = fetuses). **(B and C)** Placental (B) and fetal plasma **(C)** GDF15 levels in *Gdf15*^+/+^, *Gdf15*^+/−^ and *Gdf15*^−/−^ groups at E18.5 (*n* = 10). **(D and E)** Representative images (D) and H&E staining (E) of sagittal histological sections of fetuses at E18.5 in each group. Scale bar, 2 mm. **(F–I)** Fetal weight **(F)**, crown-rump length **(G)**, placental weight (H) and placental efficiency (I) at E18.5 in each group (*Gdf15*^+/+^, *n* = 19; *Gdf15*^+/−^, *n* = 37; *Gdf15*^−/−^, *n* = 17; *N* = 10). **(J)** Birth weight in *Gdf15*^+/+^ (*n* = 16), *Gdf15*^+/−^ (*n* = 30) and *Gdf15*^−/−^ (*n* = 17) groups (*N* = 9). **(K)** Weaning weight in *Gdf15*^+/+^ (*n* = 208), *Gdf15*^+/−^ (*n* = 385) and *Gdf15*^−/−^ (*n* = 202) groups at postnatal day 21 (*N* = 118). Data are presented as the mean ± SEM. *P* values were analyzed by one-way ANOVA with Tukey’s post hoc test. *P < 0.05, **P < 0.01, and ns (not significant). The underlying data for this figure can be found in S1 Data.

Notably, qPCR and immunohistochemical staining showed that GDF15 was highly expressed in the placenta, predominantly in the labyrinth layer, whereas remained low in various tissues (including heart, liver, lung, kidney, brain, intestine, muscle) of fetal mice during pregnancy (S3A–S3C Fig), suggesting that placenta-derived GDF15 may play a key role in fetal growth. To further investigate whether these effects are due to placental GDF15, placenta-specific *Gdf15*-deficient (*Gdf15*^PKO^, *Gdf15*^fl/fl^; *Cyp19a1*^cre/+^) mice were generated by using a *Cyp19a1*-Cre/*Gdf15-*loxP system and were identified by PCR (Fig 3A and 3B). qPCR and western blot analysis showed that CYP19A1 is highly expressed in placenta compared to fetal and maternal tissues in mice (S4A-S4B Fig). Recombinase activity was confirmed specifically in placenta but not in fetal tissues and maternal liver in mice (S4C Fig), as evidenced by crossing *Cyp19a1*-Cre with R26-LSL-EGFP reporter mice. We intercrossed *Gdf15*^fl/fl^ female mice with *Gdf15*^PKO^ male mice to generate both *Gdf15*^PKO^ and *Gdf15*^fl/fl^ conceptuses in the same pregnant mice (Fig 3C). GDF15 levels were significantly decreased in the placentas and plasma of *Gdf15*^PKO^ conceptuses compared to *Gdf15*^fl/fl^ littermates (Fig 3D–3F). However, placental GDF15 deficiency did not alter *Gdf15* mRNA expression in fetal various tissues (S5A–S5F Fig). These data indicate that placenta contributes to the fetal circulating GDF15 pool. Further analysis showed that placenta-specific GDF15 deficiency resulted in reducing fetal weight and crown-rump length at E18.5 (Fig 3G–3J), suggesting that placental GDF15 deficiency is sufficient to cause defective FGR. In addition, placental weight was lower in *Gdf15*^PKO^ conceptuses compared to *Gdf15*^fl/fl^ controls, although the difference was not statistically significant (Fig 3K). Placenta-specific GDF15 deficiency also reduced placental efficiency (Fig 3L). Together, these findings demonstrate that fetal circulating GDF15 is derived predominantly from the placenta and that placental GDF15 is essential for fetal growth in mice.

**Fig 3:**
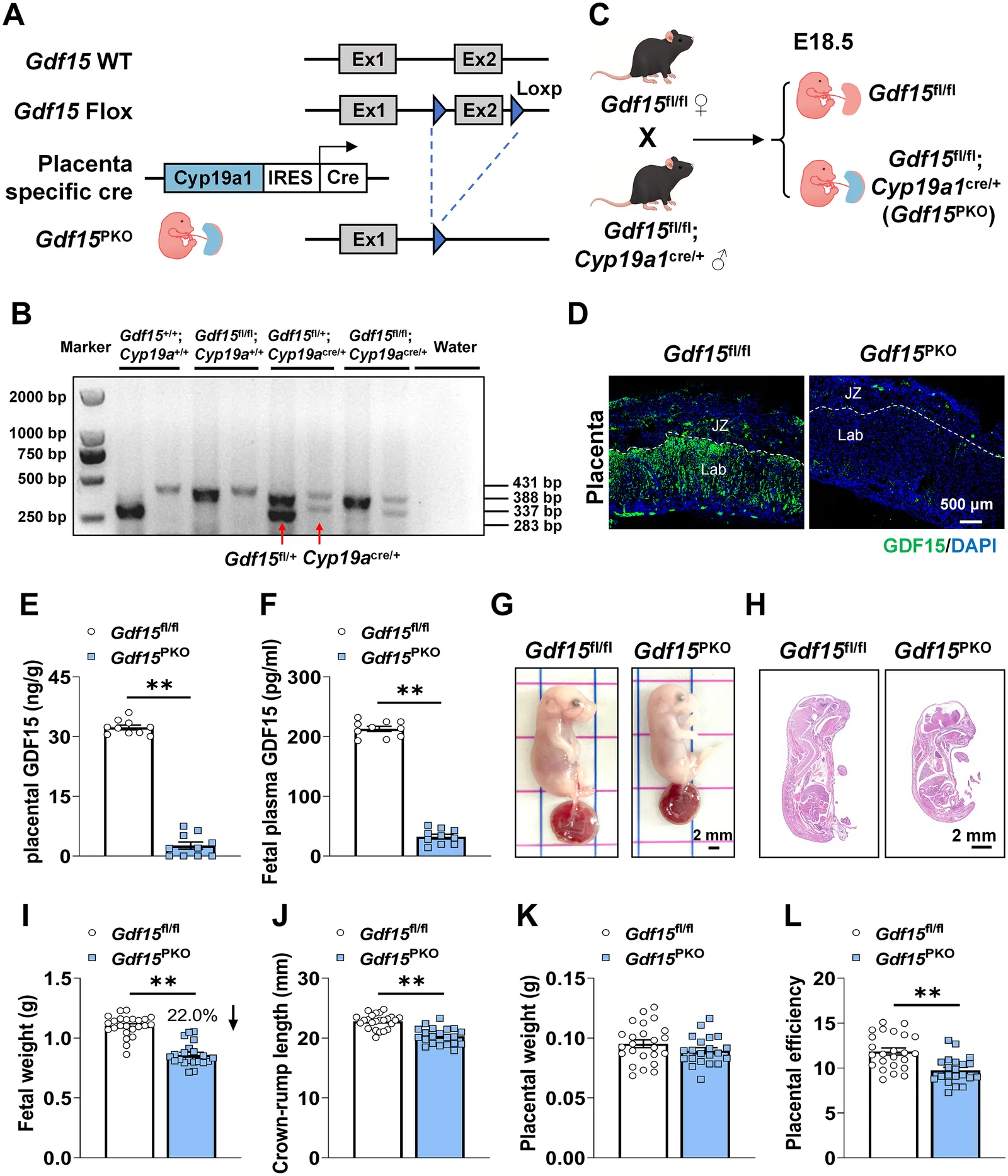
Placenta-specific GDF15 deficiency results in FGR. **(A)** *Gdf15* genetic locus before and after recombination. Exon 2 is conditionally targeted. *Cyp19a1* promoter drives expression of Cre in the placenta only. LoxP sites are indicated by blue triangles and exons 1-2 by gray boxes. **(B)** Genotype identification of *Gdf15*-flox and *Cyp19a*-Cre mice by using PCR. **(C)** Schematic diagram of breeding strategy. Generation of *Gdf15*^PKO^ (*Gdf15*^fl/fl^; *Cyp19a*^*Cre/*+^) fetuses and the littermate control (*Gdf15*^fl/fl^) fetuses using 8–10 weeks *Gdf15*^fl/fl^ female mice and *Gdf15*^PKO^ male mice. **(D)** Representative immunofluorescence staining of GDF15 (Green) in the *Gdf15*^fl/fl^ and *Gdf15*^PKO^ placentas. Scale bar, 500 μm. **(E and F)** GDF15 levels of the placentas (E) and fetal plasma (F) in *Gdf15*^PKO^ and *Gdf15*^fl/fl^ fetuses (*n* = 10). **(G and H)** Representative images (G) and H&E staining (H) of sagittal histological sections in *Gdf15*^PKO^ and *Gdf15*^fl/fl^ fetuses at E18.5. Scale bar, 2 mm. **(I–L)** Fetal weight **(I)**, crown-rump length **(J)**, placental weight (K) and placental efficiency (L) at E18.5 in each group (*Gdf15*^fl/fl^, *n* = 24; *Gdf15*^PKO^, *n* = 21). Data are presented as the mean ± SEM. *P* values were analyzed by two-tailed unpaired Student *t* test. *P < 0.05, **P < 0.01. *Gdf15*^PKO^, placenta-specific *Gdf15-*deficient mice. The underlying data for this figure can be found in S1 Data.

### Placental *Gdf15* overexpression ameliorates FGR

Based on the finding that placental GDF15 deficiency causes FGR in mice, we next evaluated the therapeutic potential of GDF15 for FGR. We employed a placenta-tropic ionizable lipid nanoparticle (LNP) system, LNP55, to generate placenta-targeted Cy5-labeled mouse *Gdf15* mRNA nanoparticles (Cy5-*Gdf15*-LNPs) as previously described [37]. Dynamic light scattering (DLS) characterization of the Cy5-*Gdf15*-LNP revealed a mean hydrodynamic diameter of 114.4 ± 4 nm and a polydispersity index (PDI) of 0.136 ± 0.013 (S6A and S6B Fig), indicating consistent formulation quality, with a high encapsulation efficiency (S6C Fig). Following intravenous (i.v.) administration of Cy5-*Gdf15*-LNPs to pregnant mice (S6D Fig), in vivo imaging revealed robust placenta-specific delivery and accumulation of Cy5-*Gdf15*-LNP (S6E and S6F Fig). Six hours after i.v. administration, *Gdf15*-LNPs (0.5 mg kg^−1^) treatment significantly elevated placental GDF15 levels (~10-fold) in pregnant mice compared with those of controls (S6G Fig), confirming efficient *Gdf15* overexpression in placenta.

We further evaluated the pharmacodynamic characteristics of different doses of *Gdf15* mRNA-LNPs in healthy pregnant mice. At 6 hours after intravenous administration, plasma GDF15 levels in fetal mice receiving doses of 0.1, 0.25, 0.5, 1, 2, and 5 mg kg^−1^ of *Gdf15* mRNA-LNP were approximately 1.2-fold, 2-fold, 5.3-fold, 11.5-fold, 27.4-fold, and 71.8-fold, respectively, compared with the control group (S6H Fig). These data demonstrate that *Gdf15* mRNA-LNP achieves dose-dependent GDF15 protein expression in the fetal circulation. Additionally, no obvious alterations in liver injury or inflammatory markers were shown in healthy pregnant mice treated with different dose (S6I–S6L Fig). Given that excessively high GDF15 during pregnancy may cause adverse effects such as nausea and vomiting [17], we assessed the safety of *Gdf15* mRNA-LNPs by measuring food intake 24 h after a single injection in FGR pregnant mice. Doses of 0–1 mg kg^−1^ did not affect food intake, whereas 2 and 5 mg kg^−1^ significantly reduced it compared with controls (S6M Fig). Accordingly, 0.5 mg kg^−1^ *Gdf15* mRNA-LNPs were selected as the therapeutic dose for subsequent experiments.

To evaluate its therapeutic effect, FGR pregnant mice received *Gdf15* mRNA-LNP treatment at E10 (Fig 4A). Compared with healthy pregnant mice (control group), FGR mice exhibited significantly decreased fetal weight and crown-rump length, as well as reduced placental and fetal plasma GDF15 levels (Fig 4B–4H). *Gdf15* mRNA-LNPs (0.5 mg kg^−1^) treatment significantly elevated GDF15 levels in the placenta and fetal plasma relative to the FGR group, but did not alter maternal plasma GDF15 levels (Figs 4B–4D and S6N). Moreover, this treatment ameliorated the FGR phenotype with increased fetal weight, crown-rump length, fetal liver weight, placental weight and placental efficiency (Fig 4E–4K), without affecting embryo number or resorption rate (Fig 4L and 4M). These results demonstrate that placenta-targeted GDF15 administration is a promising approach for treating FGR.

**Fig 4:**
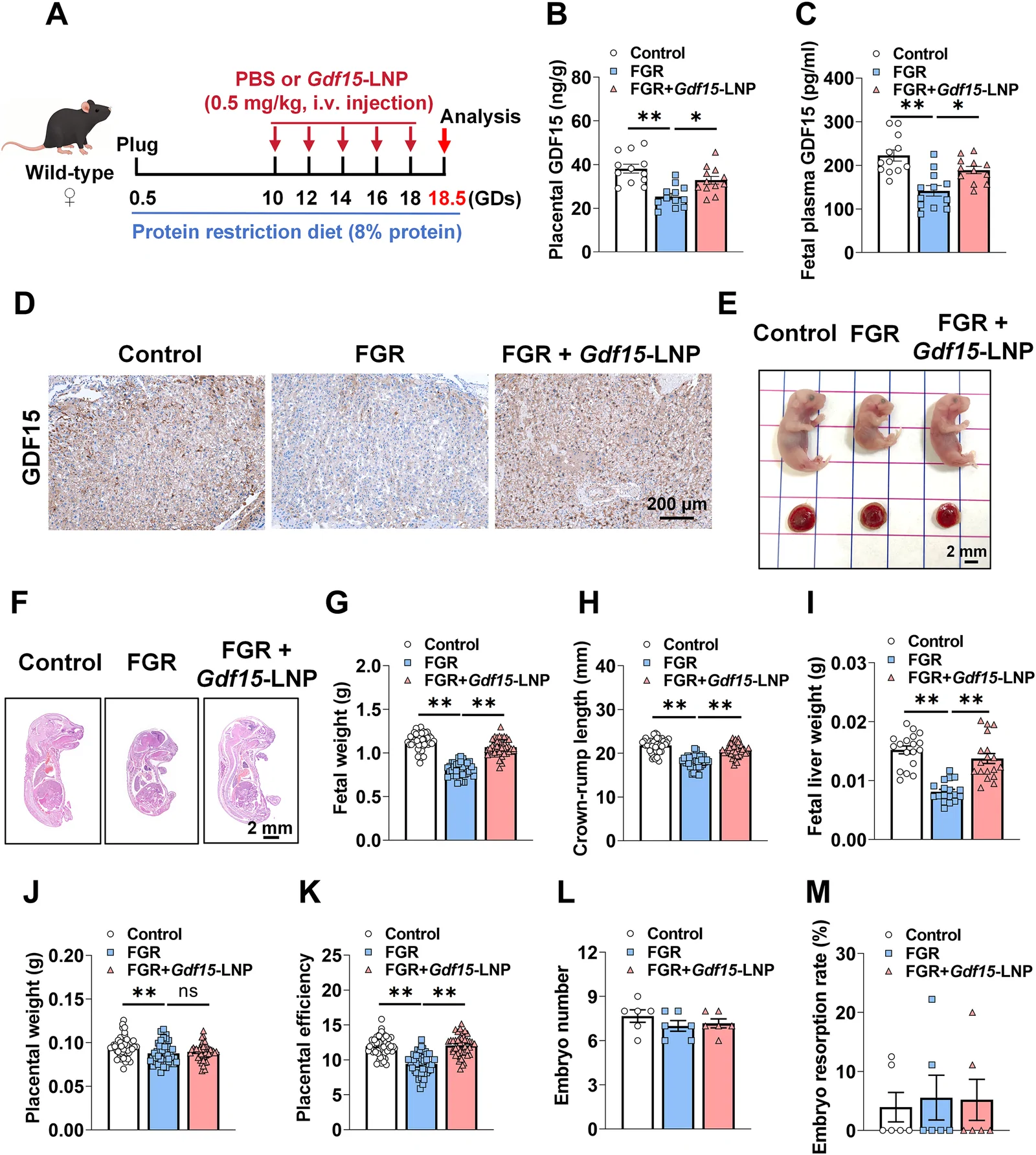
Placental *Gdf15* overexpression ameliorates FGR. **(A)** Schematic diagram of mice treatment. FGR model mice were induced by protein restriction diet (8%). Eight weeks FGR pregnant dams were treated with *Gdf15* mRNA-LNP (0.5 mg kg^−1^ maternal body weight, i.v. injection, every other day) or LNP-Luc mRNA from GD10 to GD18 (*N* = dams, *n* = fetuses). Control group in which healthy pregnant mice receiving normal diet and the same volume of PBS injection as the FGR + *Gdf15*-LNP group. **(B and C)** GDF15 protein levels of placentas (B) and fetal plasma (C) in each group at E18.5 (*n* = 12 from 6 litters). **(D)** Representative immunohistochemical staining for GDF15 in placental sections in each group. Scale bar, 200 μm. **(E and F)** Representative images (E) and H&E staining (F) of fetal sagittal histological sections at E18.5. Scale bar, 2 mm. **(G and H)** Fetal weight (G) and crown-rump length (H) at E18.5 in each group (control, *n* = 46; FGR, *n* = 42; FGR + *Gdf15*-LNP, *n* = 43). **(I)** Fetal liver weight in each group (*n* = 18). **(J and K)** Placental weight (J) and placental efficiency (K) in each group (control, *n* = 46; FGR, *n* = 42; FGR + *Gdf15*-LNP, *n* = 43). **(L and M)** Embryo number (L) and resorption rate (M) of pregnant mice at E18.5 in each group (*N* = 6 litters). Data are presented as the mean ± SEM. *P* values were calculated by one-way ANOVA with Tukey’s post hoc test. *P < 0.05, **P < 0.01. The underlying data for this figure can be found in S1 Data.

### Placenta-derived GDF15 promotes fetal growth by stimulating fetal hepatic IGF2 production

Based on the contribution of placental GDF15 to fetal circulating GDF15 and fetal growth, we speculated that it controls fetal growth in an endocrine manner. To test this, we examined whether placental GDF15 enters into the fetal circulation during pregnancy. We first employed a BeWo trophoblast monolayers as an in vitro placental barrier model [38] and the integrity of the monolayers was confirmed by the absence of fluorescein isothiocyanate (FITC)-dextran translocation from the apical to the basolateral chamber (S7A and S7B Fig). We then added recombinant mouse GDF15 (rmGDF15) to the apical chamber and found detectable rmGDF15 in the basolateral supernatant (S7C Fig), demonstrating that GDF15 is able to cross the trophoblast layer. We next validated this finding in vivo by injecting the fluorescently labeled recombinant GDF15 (Cy5.5-rGDF15) into the placenta of pregnant mice (S8A Fig). Cy5.5-rGDF15 was detectable within the placentas and fetuses (S8B Fig), confirming that Cy5.5-rGDF15 in the placenta is able to enter the fetal circulation. Consistent with this, a single administration of *Gdf15*-LNP for 6 hours significantly increased fetal plasma GDF15 levels by more than 5-fold (S6H Fig), indicating that placenta-derived GDF15 successfully secreted into fetal circulation. Notably, Cy5.5-rGDF15 preferentially accumulated in the fetal liver (S8C Fig). These data suggest that placental GDF15 can be actively transferred into the fetal circulation and reveal a potential endocrine mechanism by which placental GDF15 acts on fetal liver to promote fetal growth.

Given the specific accumulation of Cy5.5-rGDF15 in the fetal liver, we further performed proteomic analyses on liver samples from placenta-specific *Gdf15*^PKO^ fetuses and *Gdf15*^fl/fl^ control fetuses to identify the potential downstream mediator of placental GDF15 (Fig 5A). The analysis revealed 142 differentially expressed proteins, including 38 secretory proteins, significantly enriched in *Gdf15*^fl/fl^ fetal livers (fold change ≥ 2; *P* < 0.05) compared to *Gdf15*^PKO^ fetal livers (Fig 5B). Among these differentially secretory proteins, IGF2 was identified to rank top1 (Fig 5C). IGF2 has been reported as a key regulator of fetal growth [39,40] and was highly expressed in the fetal liver (Figs 5D and S9A). Further analysis showed that IGF2 protein levels were significantly downregulated in the liver and plasma of *Gdf15*^PKO^ fetuses compared to controls (Fig 5E–5H). qPCR analysis confirmed that placenta-specific GDF15 deficiency markedly decreased *Igf2* mRNA expression in fetal liver (Fig 5I). We also detected the alterations of IGF2 expression in other tissues of fetal mice and found that placental GDF15 deficiency did not significantly alter *Igf2* mRNA or protein expression in other fetal tissues, including muscle, lung and heart (S9B–S9G Fig). These results suggest that placental GDF15 deficiency specifically suppresses IGF2 expression in fetal liver.

**Fig 5:**
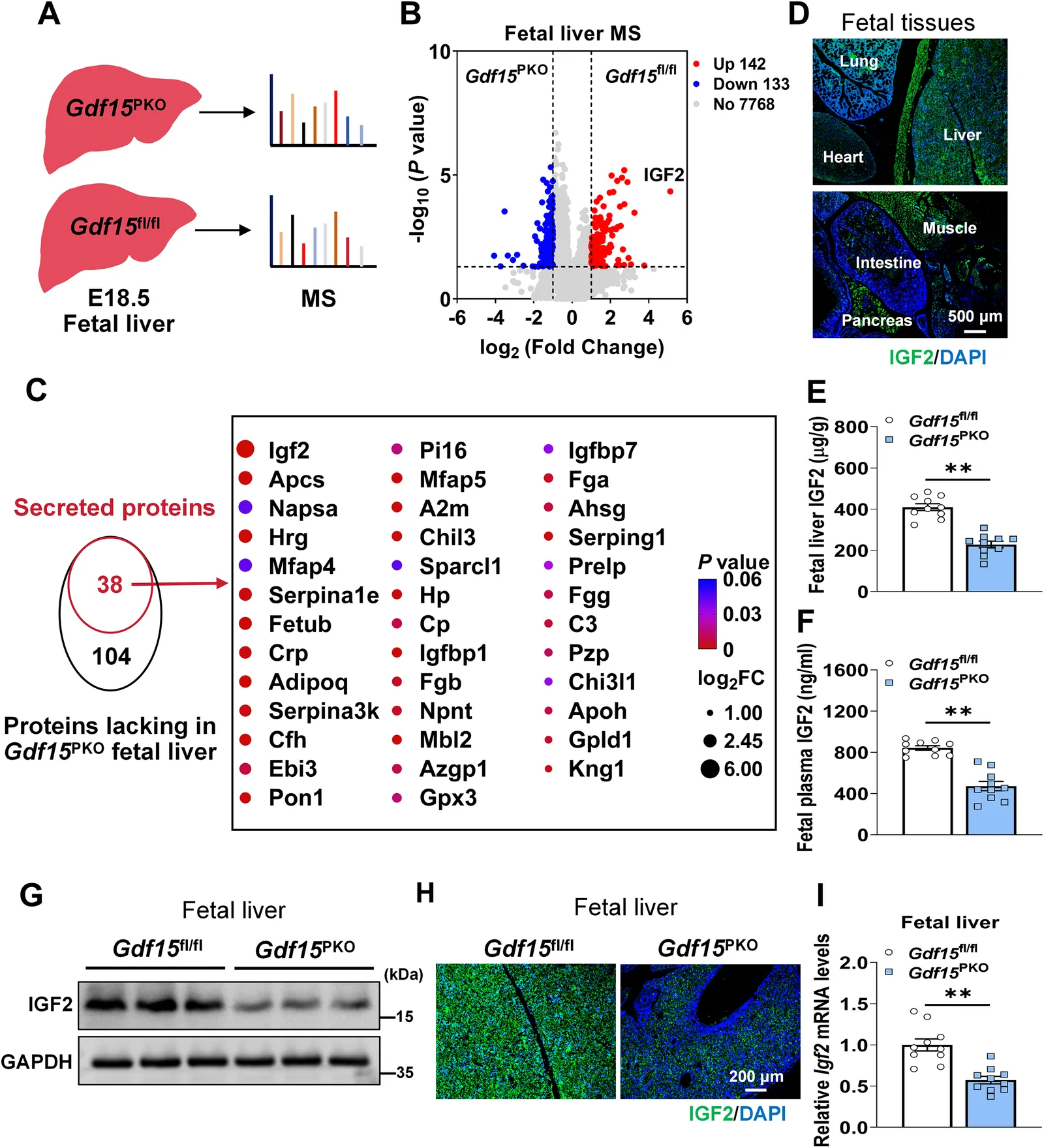
Placenta-specific GDF15 deficiency decreases hepatic IGF2 expression in fetuses. **(A)** Schematic diagram of the proteomic sequencing in the liver from *Gdf15*^PKO^ fetuses and *Gdf15*^fl/fl^ fetuses at E18.5. **(B)** Volcano plots showing differently expressed proteins in fetal liver tissues (*n* = 4). The vertical dashed lines indicate the fold change (FC) threshold (|log_2_FC| > 1), and the horizontal dashed line indicates the *P* value threshold (*P* = 0.05). **(C)** Venn diagram showing differentially expressed secretory proteins lacking in *Gdf15*^PKO^ fetal liver proteomics results (log_2_FC > 1, *P* < 0.05). **(D)** Representative immunofluorescence staining of IGF2 (Green) in fetal tissues. Scale bar, 500 μm. **(E)** ELISA analysis to quantify IGF2 levels of fetal liver in each group at E18.5 (*n* = 10). **(F)** ELISA analysis to quantify fetal plasma IGF2 levels in each group at E18.5 (*n* = 10). **(G)** Relative IGF2 protein levels of fetal liver in each group analyzed by western blot (*n* = 3). **(H)** Representative immunofluorescence staining of IGF2 (Green) of fetal liver sections in each group. Scale bar, 200 μm. **(I)** Relative mRNA expression of *Igf2* gene of liver in *Gdf15*^PKO^ and *Gdf15*^fl/fl^ fetuses (*n* = 10). Data are presented as the mean ± SEM. *P* values were analyzed by two-tailed unpaired Student *t* test. *P < 0.05, **P < 0.01. *Gdf15*^PKO^, placen*t*a-specific *Gdf15-*deficient mice; MS, mass spectrometry. The underlying data for this figure can be found in S1 Data. The Original blot for this figure can be found in S1 Raw Images.

We further analyzed the correlation between GDF15 and IGF2 during pregnancy and showed that fetal plasma IGF2 levels positively correlated with fetal plasma and placental GDF15 levels, and fetal weight at E18.5 (S10A–S10C Fig). Besides these in vivo evidences, treatment of primary mouse fetal hepatocytes with rGDF15 protein markedly upregulated *Igf2* mRNA and protein expression in a dose-dependent manner (Fig 6A and 6B). Furthermore, incubation of primary mouse fetal hepatocytes with conditioned medium from *GDF15*-overexpressing trophoblast cells also significantly increased IGF2 expression (Fig 6C–6H), demonstrating that trophoblast-derived GDF15 stimulates IGF2 expression in fetal hepatocytes. Together, these findings reveal a causal link between placental GDF15 and fetal hepatic IGF2 production.

**Fig 6:**
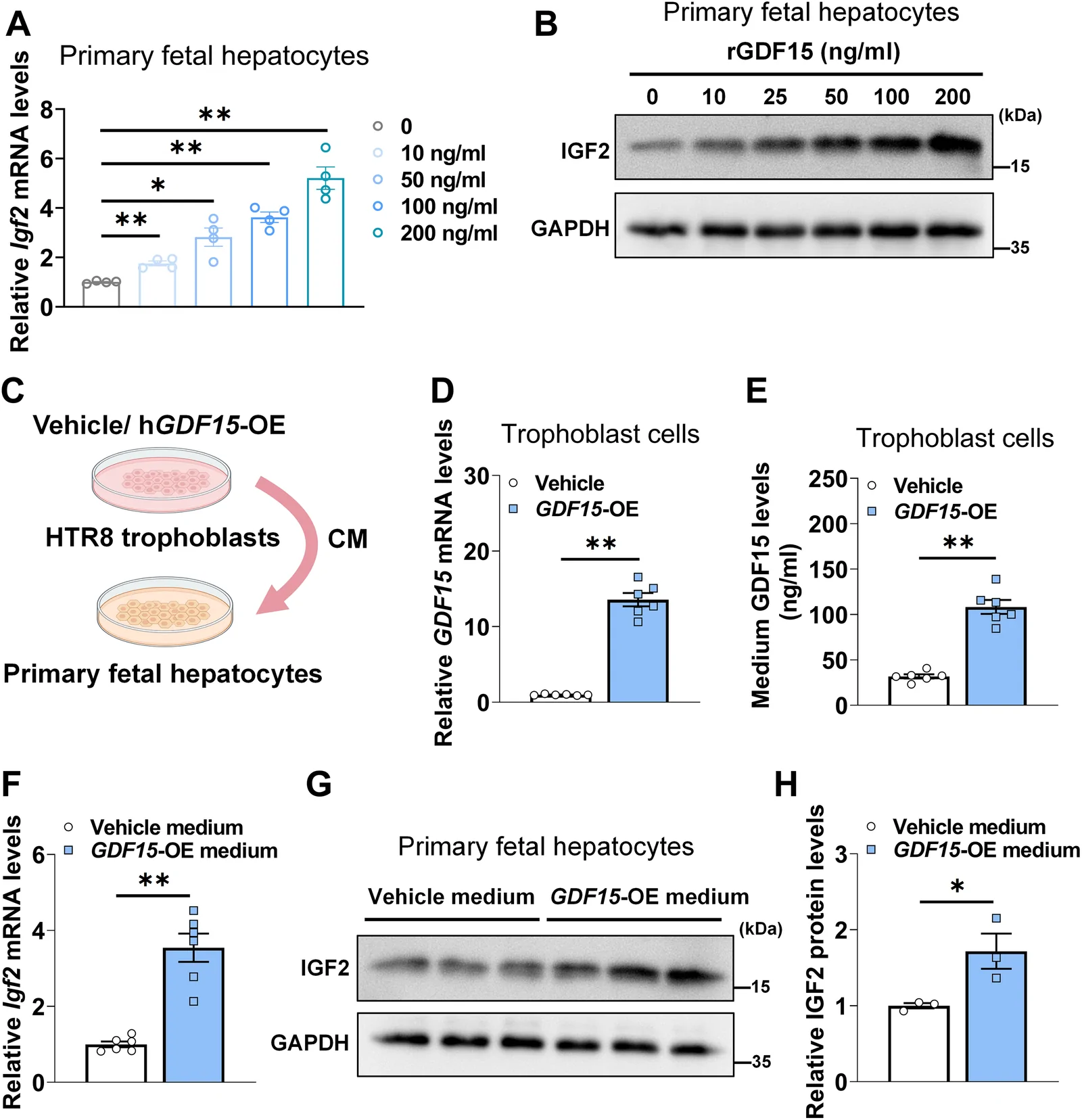
Trophoblast-derived GDF15 facilitates IGF2 expression in mouse primary fetal hepatocytes. **(A)** Relative *Igf2* mRNA expression in fetal primary hepatocytes treated with rGDF15 (0-200 ng/ml) for 24 h (*n* = 4). **(B)** Relative IGF2 protein levels in fetal primary hepatocytes treated with rGDF15 (0-200 ng/ml) for 24 **h. (C)** Schematic diagram of cell culture system. Fetal primary fetal hepatocytes were cultured with the culture medium of human *GDF15*-overexpressing vector (h*GDF15*-OE) or vehicle-treated HTR8 trophoblast cells for 24 **h. (D)** Relative *GDF15* mRNA expression in h*GDF15*-OE or vehicle-treated HTR8 trophoblast cells for 48 h (*n* = 6). **(E)** GDF15 levels of culture medium in h*GDF15*-OE or vehicle-treated HTR8 trophoblast cells (*n* = 6). **(F)** Relative *Igf2* mRNA expression in fetal primary hepatocytes treated with the culture medium of HTR8 trophoblast cells for 24 h (*n* = 6). **(G and H)** Quantification of IGF2 protein levels in fetal primary hepatocytes treated with the culture medium of HTR8 cells for 24 h (*n* = 3). Data are presented as the mean ± SEM. *P* values were analyzed by one-way ANOVA with Tukey’s post hoc test (A) or two-tailed unpaired Student *t* test **(D-F)**. *P < 0.05, **P < 0.01. rGDF15, recombinant GDF15 protein; h*GDF15*-OE, human *GDF15-*overexpressing vector; CM, culture medium. Panel C created in BioRender. liu, **X.** (2026) https://BioRender.com/ax7lt7r. The underlying data for this figure can be found in S1 Data. The Original blot for this figure can be found in S1 Raw Images.

### Placental GDF15 promotes fetal hepatic IGF2 expression by activating STAT3 signaling during fetal growth

To elucidate the underlying mechanism by which GDF15 improves fetal hepatic IGF2 expression to promote fetal growth, we further analyzed the liver proteomic data from *Gdf15*^PKO^ fetuses and *Gdf15*^fl/fl^ control fetuses. Kyoto Encyclopedia of Genes and Genomes (KEGG) enrichment analysis showed that differentially expressed proteins are markedly enriched in the JAK-STAT signaling pathway (Fig 7A). Signal Transducer and Activator of Transcription 3 (STAT3) is a major component in this pathway and has been shown to play a critical role in regulating IGF2 expression in human tumor cells [41]. We then investigated whether STAT3 is involved in placental GDF15-induced fetal hepatic IGF2 expression. Western blot analysis showed that placental GDF15 deficiency significantly inhibits STAT3 phosphorylation (p-STAT3) in fetal liver but not in fetal muscle, lung, heart, or kidney (Figs 7B and S11A–S11D), indicating that placenta-derived GDF15 specifically regulates the STAT3 signaling in the fetal liver. Additionally, rGDF15 treatment significantly increased STAT3 phosphorylation (p-STAT3) levels in primary mouse fetal hepatocytes (Fig 7C). To further establish the role of STAT3 activation in this process, STAT3 was inhibited with Stattic (2 μM), which effectively abolished rGDF15-induced STAT3 phosphorylation (Fig 7C) and blocked the increases of *Igf2* mRNA and protein expression in primary mouse fetal hepatocytes (Fig 7C and 7D). These data demonstrate that STAT3 signaling is essential for placental GDF15 to drive fetal hepatic IGF2 expression.

**Fig 7:**
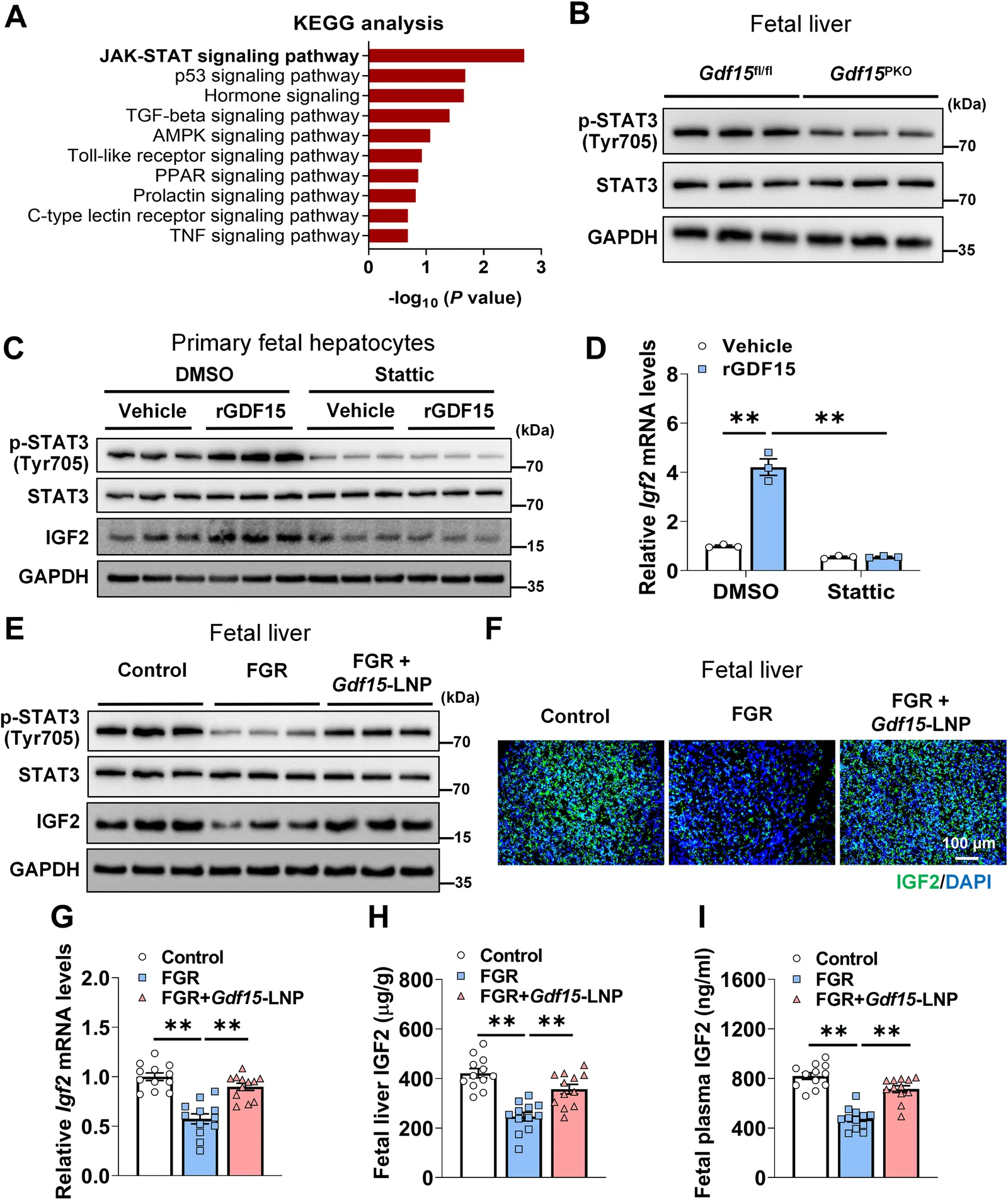
Placental GDF15 promotes fetal hepatic IGF2 expression by activating STAT3 signaling. **(A)** KEGG pathway enrichment analysis of differently expressed proteins in fetal liver. **(B)** Relative protein expression of STAT3 and p-STAT3 in *Gdf15*^PKO^ and *Gdf15*^fl/fl^ fetal livers (*n* = 3). **(C)** Relative protein levels of IGF2 and STAT3 signaling pathway in primary fetal hepatocytes pre-treated with a specific STAT3 inhibitor, Stattic (2 μM) or DMSO followed by rGDF15 (100 ng/ml) or PBS administration (*n* = 3). **(D)** Relative *Igf2* mRNA expression in primary mouse fetal hepatocytes pre-treated with DMSO or Stattic and stimulated with rGDF15 (*n* = 3). **(E)** Relative protein abundance related to IGF2 and STAT3 signaling pathway of fetal liver in each group (*n* = 3). **(F)** Representative immunofluorescence staining of IGF2 (Green) in fetal liver sections in control, FGR, FGR + *Gdf15*-LNP group. Scale bar, 100 μm. **(G and H)** The mRNA (G) and protein (H) expression of *Igf2* gene in fetal liver in control group, FGR group, and FGR + *Gdf15*-LNP-treated group at E18.5 (*n* = 12). **(I)** Fetal plasma IGF2 levels in each group, analyzed by ELISA (*n* = 12). Data are presented as the mean ± SEM. *P* values were calculated by 2-way ANOVA with Sidak’s post hoc test (D) and one-way ANOVA with Tukey’s post hoc test (G–I). *P < 0.05, **P < 0.01, and ns (not significant). The underlying data for this figure can be found in S1 Data. The Original blot for this figure can be found in S1 Raw Images.

We further analyzed how STAT3 regulates *Igf2* transcription in primary mouse fetal hepatocytes. Mouse *Igf2* regulation is highly promoter-specific, and its transcripts from promoters 2 (P2) and 3 (P3) are present in developing embryo, with dominant expression from the P3 [42–44]. A putative STAT3 binding site was shown in the P3 of mouse *Igf2* gene by using the JASPAR website (S12A Fig). Approximately 1 kb of the murine *Igf2* gene P3 region was cloned into a PGL3 luciferase reporter vector (m*Igf2*-Luc) (S12B Fig). The luciferase reporter assay showed that mutation of the putative STAT3 response element significantly decreased the luciferase activity of *Igf2* P3 (S12C Fig), whereas *Stat3* overexpression increased its luciferase activity (S12D Fig), demonstrating STAT3-driven *Igf2* gene transcription. ChIP-PCR confirmed that STAT3 directly binds to the endogenous *Igf2* promoter in primary mouse fetal hepatocytes (S12E Fig). Together, these findings establish STAT3-mediated transcriptional activation of *Igf2* in mouse fetal hepatocytes.

We further determined whether placental GDF15 regulates the fetal hepatic STAT3-IGF2 signaling in FGR mice. Compared to normal controls, FGR fetuses exhibited significantly reduced placental and fetal GDF15 levels (Fig 4B–4D), accompanied by decreased hepatic p-STAT3 and IGF2 expression (Fig 7E–7H). Administration of *Gdf15* mRNA-LNP to FGR pregnant mice markedly elevated the placental and fetal plasma GDF15 levels (Fig 4B–4D), and restored the reduced hepatic p-STAT3 and IGF2 protein levels and circulating IGF2 levels in FGR fetuses (Fig 7E–7I), and ultimately ameliorated FGR (Fig 4E–4H). Collectively, these results confirm that placenta-derived GDF15 activates fetal hepatic STAT3-IGF2 signaling axis, thereby rescuing FGR.

## Discussion

FGR is a major contributor to perinatal morbidity and mortality [2,45] and affects offspring health [3–6], its pathogenesis is poorly understood. In this study, we demonstrate that placental and fetal circulating GDF15 levels positively correlate with fetal weight in both mouse and pig models. Loss of placental GDF15 induces FGR, while placenta-specific *Gdf15* overexpression effectively ameliorates the phenotype. Mechanistically, we uncover that placental GDF15 enters the fetal circulation, where it activates the STAT3 signaling pathway to promote IGF2 expression and secretion in the fetal liver, thereby driving fetal growth. These findings uncover a previously unrecognized placenta-fetal liver endocrine regulatory axis centered on GDF15-STAT3-IGF2 signaling that governs fetal growth, greatly expanding the understanding of the biological role of GDF15 during late pregnancy. Meanwhile, our study reveals that placental GDF15 is a key factor in regulating FGR, providing a promising therapeutic target for FGR.

Previous studies have shown that decreased GDF15 protein levels of serum or placental villi in humans and mice are implicated in early miscarriage [22,23]. GDF15 supplementation ameliorated the pathological manifestations by improving placental function in lipopolysaccharide-induced abortion model mice [24], beneficial for early pregnancy maintenance. However, recent findings have reported that the excessively elevated GDF15 of the maternal circulation caused by GDF15 variant (C211G) in humans increases the risk of developing nausea and vomiting or hyperemesis gravidarum in early pregnancy [17,46], suggesting that circulating GDF15 inhibition might be a promising approach for pregnancy-associated vomiting. Here, our studies have emphasized the protective role of GDF15 in FGR. We demonstrated that placental and fetal circulating GDF15 levels strongly link to the risk of FGR in mice and pigs, consistent with previous clinical observations showing decreased plasma GDF15 in small-for-gestational-age patients whose neonates with low birthweight during late pregnancy [25]. More importantly, we revealed that placenta-specific *Gdf15* mRNA-LNP delivery in mid-to-late gestation effectively rescued FGR, strongly expanding the key role of GDF15 in late pregnancy. These findings underscore the multifunctional role of GDF15 and its significant clinical translational potential across different pregnancy complications. In particular, our study achieved a “precise supplementation” of GDF15 by using placenta-specific mRNA-LNP delivery technology. This targeted approach might avoid some potential adverse effects such as nausea and vomiting of pregnancy caused by systemic GDF15 administration, thereby establishing a safe and effective intervention strategy for patients with FGR.

GDF15 is classically a stress signal. Under pathological conditions such as cancer, hypoxia, and inflammation, circulating GDF15 rises markedly [47] and acts mainly on brainstem GFRAL-RET to induce anorexia and weight loss [48,49]. In contrast, under physiological conditions, GDF15 can exert beneficial effects. Exercise-induced skeletal muscle GDF15 acts on pancreatic islets to enhance glucose-stimulated insulin secretion independently of GFRAL [50,51]. Moreover, GDF15 promotes trophoblast invasion and differentiation to support early pregnancy [23,52]. During human pregnancy, placental GDF15 elevates maternal circulating levels and serves as a physiological signal for maternal metabolic adaptation [17]. In early pregnancy, a moderate increase in placental GDF15 may induce mild nausea and vomiting, potentially conferring an evolutionary advantage by protecting the embryo from dietary toxins, and in mid-to-late pregnancy, progressive GDF15 elevation contributes to maternal metabolic adaptation and fetal growth. In our study, fetal circulating GDF15 is predominantly derived from the placenta during pregnancy in mice. With adequate placental function and nutrient supply, placenta-derived GDF15 enters the fetal circulation and acts on the fetal liver to activate STAT3 signaling, promoting IGF2 expression and fetal growth. Conversely, placental dysfunction or nutrient insufficiency reduces placental GDF15, leading to decreased fetal circulating GDF15, suppressed hepatic STAT3-IGF2 signaling, and FGR. Thus, placental GDF15 links placental function to fetal hepatic growth signaling. Collectively, these findings suggest that elevated placental GDF15 during pregnancy represents a physiological adaptive signal rather than a classical pathological stress signal, with target-organ specificity: it participates in maternal metabolic adaptation and regulates fetal growth via the STAT3-IGF2 axis on the fetal side.

Our findings demonstrate that placental and fetal plasma GDF15 levels positively correlate with fetal weight in mouse and pig models, and that either systemic or placenta-specific GDF15 deficiency causes FGR. A previous study reported that human GDF15 loss-of-function carriers with C211G homozygous remain fertile and have multiple children [53]. However, this study did not report birth weight, placental phenotypes, FGR diagnosis, or combined maternal-fetal genotypes [53]. Thus, we cannot conclude whether GDF15 mutations influence fetal growth in humans, which represents a critical knowledge gap. Of note, recent human studies on hyperemesis gravidarum-related genetic loci have revealed that GDF15 variant rs1058587 exerts opposite effects on HG risk between the maternal and fetal genotypes, with maternal genotype influencing prepregnancy GDF15 baseline and fetal genotype modulating placental GDF15 production, jointly increasing HG risk [17,54]. However, no human study has directly assessed combined maternal-fetal GDF15 genotypes in relation to fetal growth. Here, our reciprocal crossing experiments showed that maternal GDF15 deficiency did not alter fetal circulating GDF15 levels or body weight, whereas placenta-specific GDF15 deficiency markedly reduced fetal circulating GDF15 and caused FGR. These results indicate that fetal circulating GDF15 is largely derived from the placenta and that its GDF15 on fetal growth is primarily determined by the fetal/placental genotype rather than the maternal genotype in mice. Whether this holds in humans remains unclear. Future large-scale prospective cohort studies with paired maternal and fetal genotypes and detailed fetal growth measurements will be needed to determine whether the findings in humans are consistent with those in mice and pigs.

FGR is a complex, multifactorial disorder involving maternal factors, placental factors, and fetal factors [55,56]. Among the diverse mechanisms underlying FGR, the placenta serves as a critical bridge between the mother and fetus, and its dysfunction constitutes a key pathological basis of the disorder [56]. A recent study in pregnant sows has revealed that insufficient leucine transport across the placental trophoblast at the maternal-fetal interface leads to a reduction in specific type II muscle fiber subsets and contributes to FGR [9]. Moreover, several maternal cytokines (e.g., FGF21 [57], SERPINA3C [38]) and placental factors (e.g., exosomal PPARγ [58], SOD3 [59]) have been shown to exert endocrine effects on fetal growth or metabolic health. Our findings uncover a novel placenta-fetal liver metabolic axis: the GDF15-STAT3-IGF2 axis, as an important component of placental secretory communication, further expands the regulatory network of fetal growth. Placenta-derived GDF15, as an endocrine signal, enters the fetal circulation, activates hepatic STAT3 signaling, and stimulates IGF2 production, a key fetal growth regulator essential for matching placental microvasculature expansion to fetal growth [60]. In the protein restriction diet-induced FGR mice, reduced placental GDF15 led to decreased hepatic IGF2 levels and subsequent growth restriction. LNP-targeted restoration of placental GDF15 reactivated endogenous STAT3/IGF2 signaling and drove fetal growth. Moreover, GDF15 may improve placental function by promoting trophoblast invasion and differentiation [23,52], indirectly supporting fetal growth. Therefore, the rescue effects of placenta-targeted *Gdf15* mRNA-LNPs likely reflect the coordinated restoration of fetal endocrine signaling and placental function, deepening our understanding of the role of placenta in regulating fetal growth.

IGF2 is a key fetal growth-promoting factor whose expression is subject to multilayered regulation, including epigenetic and transcriptional mechanisms. Epigenetically, the H19/IGF2 imprinting control region governs paternal allele-specific IGF2 expression via DNA methylation and CTCF-dependent insulation. Its disruption causes Silver–Russell syndrome or Beckwith–Wiedemann syndrome [61,62]. Transcriptionally, the HMGA2-PLAG1-IGF2 axis promotes IGF2 expression, and its disruption is linked to FGR [63], C/EBP-β similarly activates *Igf2* gene P3 promoter in mouse fetal liver [64]. Beyond cell-autonomous regulation, placenta-derived signals also modulate fetal hepatic IGF2 expression. It was shown that SPAG7 deficiency causes placental insufficiency and secondary reductions in fetal hepatic IGF2 [65]. However, the placental signaling molecule responsible for IGF2 regulation remains unclear. Here, we identify GDF15 as a direct endocrine signal from the placenta to the fetus. We further demonstrate that placenta-derived GDF15 activates STAT3 signaling in the fetal liver, thereby promoting *Igf2* P3 promoter activity and its expression. Meanwhile, the specific inhibition of STAT3 effectively blocked GDF15-induced IGF2 upregulation in primary fetal hepatocytes, similar with previous finding that STAT3 promotes IGF2 expression in tumor cells [41]. More importantly, placenta-specific *Gdf15* mRNA-LNPs treatment rescued the FGR phenotype by restoring the hepatic STAT3-IGF2 signaling in fetuses. These findings extend the regulatory network of IGF2 expression beyond genomic imprinting and tissue-intrinsic transcriptional regulation to include endocrine crosstalk between the placenta and fetal organs, thereby providing a new cross-tissue perspective for understanding the pathogenesis of FGR.

Several limitations of this study should be acknowledged. Although we confirmed that GDF15 can be transported into the fetal circulation, however, the exact transfer mechanism remains unclear and may involve transcytosis or a specific transporter.

In summary, our study establishes placenta-derived GDF15 as a critical regulator of fetal growth that acts through the hepatic STAT3-IGF2 signaling axis. These findings uncover a novel molecular mechanism underlying FGR and identify GDF15 as a promising therapeutic target for this pregnancy complication.

## Materials and methods

### Animals

All animal procedures followed the Guide for the Care and Use of Laboratory Animals (Eighth Edition) and were approved by the Northwest A&F University Animal Care and Use Ethics Committee (approval ID: IACUC2025-0517). Male and female C57BL/6J mice were obtained from Xi’an Jiao Tong University and housed under standard conditions (12-h light/dark cycle, 23 ± 2 °C, 50 ± 10% humidity), with ad libitum food and water. Mice were randomly assigned to experimental groups. Newborn piglets were obtained from pregnant sows (Yorkshire × Landrace) at the Shaanxi Jingyang Xingao Laozhuang Breeding Co. (Xianyang, China). All pregnant sows (2–4 parities) were individually housed in individual gestation stalls (2.20 × 0.64 m) under controlled environmental conditions (mean temperature, 20 °C; relative humidity, 60%−70%) maintained by an automatic ventilation system. The room temperature of the lactating barn was kept at 28 °C ± 2 °C, and the baby house under a heating lamp was kept at 32 °C ± 2 °C, both automatically regulated. After parturition, piglets were suckled by the sows.

### Experimental design

FGR mouse models were established as previously described [6]. Briefly, C57BL/6J pregnant mice were fed with low-protein diet (8% protein, normal diet for 20% protein) to construct the protein restriction diet-induced FGR (PR-FGR) mouse model. The Dex-FGR model was induced by subcutaneous injection with dexamethasone (HY-14648, MedChemExpress, USA) (2 mg kg^−1^, every other day) from E11 to E17. Pregnancy was confirmed by vaginal plug (E0.5). At E18.5, pregnant mice were euthanized by isoflurane inhalation. The growth parameters (fetal weight and crown-rump length) and embryo absorption rate were recorded. Placental efficiency was calculated as the ratio of fetal weight to placental weight (g/g). Placentas were weighed after removal of membranes and umbilical cord and blotting of surface fluid, and each fetus was paired with its corresponding placenta. Blood was centrifuged (1,000*g* for 15 min at 4 °C) to obtain plasma. Placentas and fetuses were collected via cesarean section, snap-frozen in liquid nitrogen, or fixed in 4% paraformaldehyde for subsequent analyses. For fetal biochemical measurements (fetal plasma GDF15 and IGF2), we randomly sampled one male and one female fetus per litter, maintaining a 1:1 ratio to minimize potential confounding by sex. For growth phenotypes, all fetuses per litter were included. For FGR piglet model, mean birth weight of piglet was 1.42 ± 0.201 kg (mean ± SD). Piglets with birth weight 2 SD below the mean were defined as FGR, and those between 1.3 and 1.7 kg as normal. From these cohorts, 18 pairs of normal and FGR piglets were selected, ensuring one male and one female from each litter were chosen. Placentas located 3–4 cm from the umbilical cord insertion and umbilical vein blood samples for piglets were collected for subsequent analyses.

### Generation of *Gdf15* gene-edited mice

*Gdf15*-knockout (*Gdf15*^−/−^) mice (S-KO-07014) and *Cyp19a1*-IRES-Cre mice (C001382) were obtained from Guangzhou Cyagen Biosciences. The IRES-Cre is inserted into the termination codon downstream of endogenous *Cyp19a1* gene. *Gdf15*^fl/fl^ mice were donated by Meng Zhou’s lab from Anhui Medical University. Eight to ten weeks female *Gdf15*^+/−^ mice were obtained by heterozygote breeding. *Gdf15*^fl/fl^ mice and *Cyp19a1*-Cre mice were used to generate placenta-specific *Gdf15*-deficient (*Gdf15*^PKO^, *Gdf15*^fl/fl^; *Cyp19a*^Cre/+^) mice. *Gdf15*^PKO^ fetuses and littermate controls (*Gdf15*^fl/fl^) were acquired by crossing *Gdf15*^fl/fl^ female mice with *Gdf15*^PKO^ male mice. R26-CAG-LSL-Luc-2A-EGFP mice (NM-KI-190095) were purchased from Shanghai Model Organisms Center Recombinase activity was measured by crossing Cyp19-Cre with R26R-EYFP reporter mice. Genotyping was performed by PCR (primers listed in S1 Table).

### Placenta-tropic Cy5-*Gdf15* mRNA-LNP55 production and characterization

Placenta-targeting ionizable lipids (LNP55 formulation) were obtained from APEx Biological Co., LTD in accordance with the LNP55 formulation as previously described [37]. Mouse *Gdf15* mRNA was synthesized and capped by in vitro transcription from a double-stranded DNA template (Qingke Biological Co.) using T7 Co-transcription Kit with CAG Trimer (DD4203, Vazyme, China, Nanjing). *Gdf15* mRNA was labeled with Cy5 fluorescent dye (APEx Biological, USA). Cy5-labeled *Gdf15* mRNA was formulated into LNP55 at a 10:1 molar ratio of ionizable lipid to mRNA by mixing ethanol and aqueous phases using pipette, dialyzed against 1× PBS for 4 h using Pierce microdialysis plates. *Gdf15* mRNA-LNPs were dialyzed in the Pierce microdialysis plates (A50472, Thermo Fisher Scientific, USA) using 1× PBS solution for 4 h. LNPs were sterilized by 0.22 µm filters and stored at 4 °C for further experiment. RNA encapsulation efficiencies of LNPs were measured by using a Quant-iT-RiboGreen RNA Quantitative kit (R32700, ThermoFisher Scientific, USA) as the manufacturer’s instructions. The size and PDI of LNPs were determined by using a Malvern Zetasizer Nano ZSP (Malvern, UK).

### Treatment of pregnant mice with *Gdf15* mRNA-LNP

For tracking in vivo, pregnant mice at E15.5 were intravenously injected with fluorescent *Gdf15* mRNA-LNP (0.5 mg kg^−1^ body weight). Six hours later, mice were euthanized; organs, placentas, and fetuses were imaged using 3D optical Living Imaging system (IVIS Spectrum, USA). To evaluate the pharmacodynamic characteristics of *Gdf15* mRNA-LNP, healthy pregnant mice were intravenously injected with different doses of *Gdf15* mRNA-LNP (0.1, 0.25, 0.5, 1, 2, and 5 mg/kg) or LNP-Luc mRNA (5 mg/kg). At 6 hours after injection, fetal blood was collected and GDF15 levels were measured. To assess the role of placental *Gdf15* overexpression, FGR pregnant mice were injected intravenously with *Gdf15* mRNA-LNP (0.5 mg kg^−1^) or LNP-Luc mRNA every other day from E10. Control group in which healthy pregnant mice receiving normal diet and the same volume of PBS injection as the FGR + *Gdf15*-LNP group. At E18.5, mice were euthanized, and samples were collected and growth parameters were analyzed.

### Intraplacental injection of fluorescent recombinant GDF15

Intraplacental injection of E17 pregnant mice was performed as described previously [66]. Cy5.5-labeled recombinant GDF15 (5 μg/10 μl, 8944-GD, R&D Systems, USA) or free Cy5.5 (produced by Qiangyao Biotechnology, Shanghai, China) was injected into the placenta. The incision was sutured and mice were euthanized with isoflurane after 3 h; fetuses and placentas were collected and imaged using Living Image software.

### HTR8 cell culture and treatment

HTR8/SVneo trophoblast cells (gifted by Xifeng Xiao’s lab, Air Force Medical University, Shaanxi, China) were cultured in DMEM/F-12 medium (supplemented with 10% FBS (Z7186FBS-500, ZETA LIFE, USA) and 1% penicillin/streptomycin in 37 °C incubator with 5% CO_2_. Cells were transfected with His-tagged human GDF15 plasmids or vehicle using Lipofectamine 3000 (L3000001, Invitrogen, California, USA) for 48 h.

### BeWo cell culture and treatment

BeWo cells (CCL-98, ATCC, USA) were maintained in Ham’s F-12K medium (21127022, Gibco, USA) with 10% FBS at 37 °C and 5% CO_2_. For placental barrier assay in vitro, FITC-dextran (46944, Sigma, USA) or rmGDF15 (8944-GD, R&D Systems, USA) was added to the apical chamber and were incubated for 6 h. Supernatants from the basolateral chambers were collected for further analysis.

### Primary mouse fetal hepatocytes culture and treatment

Primary mouse fetal hepatocytes were isolated as previously described [66] and cultured in high-glucose DMEM (H30022.01, HyClone, USA) with 10% FBS and 1% penicillin/streptomycin. Cells were treated with different dose rmGDF15 (0–200 ng/ml, TMPK-01244, TargetMol, USA) for 24 h. Hepatocytes were cultured with supernatant from h*GDF15*-OE plasmid-treated HTR8 cells for 24 h. STAT3 phosphorylation was inhibited using 2 μM Stattic (HY-13818, MedChemExpress, USA).

### Enzyme-linked immunosorbent assay (ELISA)

GDF15 levels were measured using mouse GDF15 ELISA (MGD150, R&D Systems, USA), pig GDF15 ELISA (SEKP-0034, Solarbio, China), and human GDF15 ELISA (DY957, R&D Systems, USA). IGF2 levels were determined using a mouse IGF-2 ELISA kit (SEKM-0285, Solarbio, Beijing, China) according to the manufacturer’s instructions.

### Plasmid construction

The coding sequences of human *GDF15* or mouse *Stat3* were cloned into pcDNA3.1 for overexpression. The mouse *Igf2* promoter was inserted into pGL3-Basic (firefly luciferase, Promega), with pRLTK (Renilla luciferase) as internal control. A mutated STAT3-binding site within the *Igf2* promoter was synthesized by Qingke Biotechnology (Beijing, China).

### Luciferase reporter assay and ChIP-qPCR

Luciferase reporter assay and ChIP-qPCR were performed as previously described [67]. Primary mouse fetal hepatocytes were transfected with mouse *Igf2* promoter luciferase reporters (WT or mutant), pcDNA3.1-*Stat3*, and pRLTK (50 ng) using Lipofectamine 3000. After 48 h, luciferase activity was measured using the Dual-Luciferase Reporter Assay System (Promega). ChIP-qPCR was conducted with STAT3 or IgG antibody according to manufacturer’s protocol (ab500, Abcam, UK). Primer sequences are listed in S2 Table.

### Real-time quantitative reverse transcription PCR (RT-qPCR) and western blotting analyses

RT-qPCR and western blotting were performed as previously described [68]. The relative gene expression level was calculated using the comparative Ct method (2^−ΔΔCt^) and quantified by normalization to *GAPDH*. Primer sequences for all genes are listed in S2 Table. The target protein was normalized to GAPDH. Antibody information for all proteins is listed in S3 Table.

### Mass spectrometry (MS) proteomics analysis

E18.5 fetal liver samples from *Gdf15*^PKO^ and *Gdf15*^fl/fl^ fetuses were collected for proteomics analysis in Novogene Bioinformatics Co. (Beijing, China). Protein lysates were centrifuged at 13,000g using YM-10 filters, reduced with 10 mM DTT (56 °C, 30 min), alkylated with 20 mM iodoacetamide (1 h, dark), and digested overnight with trypsin (50:1, 37 °C). Tryptic peptides were desalted on C18 resin column and analyzed by UHPLC coupled with DIA-MS. Differentially expressed proteins were defined as | log_2_ (fold change) | ≥ 1 and *P* < 0.05. The false discovery rate was set to <1%. The MS proteomics data have been submitted to the ProteomeXchange consortium. MS proteomics data have been uploaded to iProX (https://www.iprox.cn/page/home.html) under the dataset identifier PXD084012.

### Hematoxylin and eosin, immunofluorescence, and immunohistochemical staining

Hematoxylin and eosin and immunofluorescence staining were performed as previously described [68] according to standardized procedures. The whole-slide digital images were collected with a Pannoramic DESK Scanner (P-MIDI, P250, 3D HISTECH, Hungary). The fetal tissues samples were labeled with GDF15 or IGF2 antibodies. Nuclei were stained with DAPI (ID22502, dilution 1:1000, Solarbio, Beijing, China). Immunohistochemical staining was performed as previously described [69]. The placental samples were labeled with anti-mouse GDF15 antibodies (sc-515675, Santa Cruz, USA). Images were collected with BioTEK gen5 Software (Agilent Technologies, USA).

### Statistical analysis

Data are presented as mean ± SEM. Statistical comparisons between two groups were performed using two-tailed unpaired Student *t* test. For multiple groups, one-way ANOVA with Tukey’s post hoc test or two-way ANOVA with Sidak’s post hoc test was used. Correlation was assessed by two-tailed Pearson correlation (95% CI). All analyses were conducted using GraphPad Prism 9 software (La Jolla, CA, USA). Linear mixed-effects model (LMM) analyses were performed in R (version 4.3.1) using the lme4 and lmerTest packages and were applied to analysis fetal body weight and crown-rump length across all groups, adjusting for fetal sex. Dam ID was included as a random intercept to account for clustering of fetuses within litters, while treatment, and fetal sex were included as fixed effects. Significance was defined as *P < 0.05; **P < 0.01. The n number and statistical methods used for each analysis were indicated in figure legends.

## Supporting information

S1 Fig The GDF15 levels of placenta and fetal plasma and fetal growth phenotype in FGR pregnant mice. **(A)** Quantification of crown-rump length at E18.5 in normal (*n* = 42) and PR-induced FGR fetuses (*n* = 39). **(B and C)** Body weight (B) and crown-rump length (C) at E18.5 in normal (*n* = 25) and Dex-induced FGR fetuses (*n* = 21). **(D and E)** Representative images (D) and H&E staining of fetal sagittal histological sections (E) in normal and PR-induced FGR groups at E18.5. Scale bar, 2 mm. **(F)** Fetal liver weight in normal and PR-induced FGR groups (*n* = 15 fetuses from 5 litters). **(G and H)** Placental weight (G) and placental efficiency (H) in normal (*n* = 42) and PR-induced FGR groups (*n* = 39). **(I and J)** Placental weight (I) and placental efficiency (J) in normal (*n* = 25) and Dex-induced FGR groups (*n* = 21). **(K and L)** Embryo number (K) and resorption rate (L) in normal and PR-induced FGR dams (*N* = 5 dams). **(M)** ELISA analysis to quantify placental GDF15 levels in normal and Dex-induced FGR groups (*n* = 9 placentas from 3 litters). **(N)** ELISA analysis to quantify fetal GDF15 levels in normal and Dex-induced FGR groups (*n* = 6 from 3 litters). **(O)** GDF15 concentrations of maternal plasma in normal and PR-induced FGR groups (*N* = 5 dams). **(P)** GDF15 concentrations of maternal plasma in normal and Dex-induced FGR groups (*N* = 3 dams). Data are presented as the mean ± SEM. *P* values were calculated by two-tailed unpaired Student *t* test. *P < 0.05, **P < 0.01, and ns (not significant). Dex, dexamethasone. The underlying data for this figure can be found in S1 Data.
(TIF)

S2 Fig Maternal GDF15 deficiency has no effect on fetal growth. **(A)** Schematic diagram for the generation of *Gdf15*-deficient mice using CRISPR/Cas9 technology. **(B)** Genotype identification of *Gdf15*^+/+^, *Gdf15*^+/−^, *Gdf15*^−/−^ mouse by PCR. **(C)** Schematic diagram of breeding strategy in which WT (*Gdf15*^+/+^) female mice × *Gdf15*^−/−^ male mice and the *Gdf15*^−/−^ female mice × WT male mice (*N* = dams, *n* = fetuses). **(D)** Fetal plasma GDF15 levels in WT and *Gdf15*^−/−^ groups at E18.5 (WT, *n* = 18; *Gdf15*^−/−^, *n* = 20). **(E)** Representative images of fetuses at E18.5 in each group. Scale bar, 2 mm. **(F and G)** Fetal weight (F) and crown-rump length (G) at E18.5 in each group (WT, *n* = 63; *Gdf15*^−/−^, *n* = 69). **(H and I)** Embryo number (H) and resorption rate (I) of pregnant mice at E18.5 in each group (WT, *N* = 9; *Gdf15*^−/−^, *N* = 10). Data are presented as the mean ± SEM. *P* values were analyzed by two-tailed unpaired Student *t* test. *P < 0.05, **P < 0.01. The underlying data for this figure can be found in S1 Data.
(TIF)

S3 Fig Tissue distribution of GDF15 in mice during pregnancy. **(A)** Relative *Gdf15* mRNA expression measured by qRT-PCR in the uterus, placenta, and fetal tissues in pregnant female at E18.5 (*n* = 6). **(B and C)** Representative GDF15 immunohistochemistry staining in placentas (B) and fetal mice (C) at E18.5. Scale bar, 2 mm and 500 μm. Data are presented as the mean ± SEM. *P* values were calculated by one-way ANOVA with Tukey’s post hoc test (A). *P < 0.05, **P < 0.01. JZ, Junction layer; Lab, Labyrinth layer. The underlying data for this figure can be found in S1 Data.
(TIF)

S4 Fig Tissue distribution of CYP19A1 in mice during pregnancy. **(A)** Relative mRNA expression of *Cyp19a1* in various tissues of fetuses and pregnant mice at E18.5 (*n* = 6). **(B)** western blot analysis of CYP19A1 protein abundance in various tissues of fetuses and pregnant mice at E18.5. **(C)** Representative immunofluorescence images of *Cyp19a1*-Cre: R26-EGFP in fetal tissues, placenta, and adult liver. Scale bar, 2 mm and 500 μm. *P* values were analyzed by one-way ANOVA with Tukey’s post hoc test. *P < 0.05, **P < 0.01. The underlying data for this figure can be found in S1 Data. The Original blot for this figure can be found in S1 Raw Images.
(TIF)

S5 Fig Placental GDF15 deficiency does not alter *Gdf15* mRNA expression in fetal tissues. **(A–F)** Relative *Gdf15* mRNA expression in fetal liver (A), muscle (B), lung (C), heart (D), brain (E), and intestine (F) from *Gdf15*^fl/fl^ and *Gdf15*^PKO^ fetuses (*n* = 6). Data are presented as the mean ± SEM. *P* values were analyzed by two-tailed unpaired Student *t* test. *P < 0.05, **P < 0.01. The underlying data for this figure can be found in S1 Data.
(TIF)

S6 Fig Cy5-*Gdf15* mRNA-LNP specifically targets to the placenta. **(A–C)** Mean hydrodynamic diameter (A), polydispersity index (B) and encapsulation efficiency (C) of *Gdf15* mRNA-LNPs (*n* = 5). **(D)** Schematic diagram of pregnant mice treatment. 8−10 weeks pregnant dams were administrated by i.v. injection with Cy5-*Gdf15*-LNP or PBS. **(E)** Fluorescence imaging of dissected tissues in pregnant mice 6 h after i.v. injection of Cy5-*Gdf15*-LNP or PBS in vivo. **(F)** Fluorescence imaging of placentas and fetuses 6 h after i.v. injection of Cy5-*Gdf15*-LNP or PBS in vivo. Radiance efficiency scales reflect photon emission intensity (photons s^−1^ cm^−2^ sr^−1^ μW^−1^ cm^2^). **(G)** Placental GDF15 levels of pregnant mice 6 h after i.v. injection of Cy5-*Gdf15*-LNP or LNP-Luc mRNA in vivo (*n* = 6). **(H)** Fetal plasma GDF15 levels, 6h following i.v. administration of various doses of *Gdf15*-LNP or LNP-Luc mRNA to healthy pregnant mice (*n* = 6). **(I–L)** Plasma levels of alanine transaminase (ALT) (I), aspartate aminotransferase (AST) (J), tumor necrosis factor alpha (TNF-α) (K), and interleukin-6 (IL-6) (L), 6h following i.v. administration of various doses of *Gdf15*-LNP to pregnant mice (*n* = 6). **(M)** Cumulative 24-h food intake after i.v. administration of various doses of *Gdf15*-LNP or LNP-Luc mRNA to pregnant mice (*n* = 6). **(N)** Maternal GDF15 levels in pregnant mice at E18.5 in each group (*n* = 6). Data are presented as the mean ± SEM. *P* values were analyzed by two-tailed unpaired Student *t* test (G) or one-way ANOVA with Tukey’s post hoc test (H-N). *P < 0.05, **P < 0.01. Cy5-*Gdf15*-LNP, Cy5-labeled *Gdf15* mRNA lipid nanoparticle; i.v., intravenous injection; F, fetus; P, placenta; H, heart; Li, liver; S, spleen; Lu, lung; K, kidney. Panel D created in BioRender. liu, X. (2026) https://BioRender.com/ax7lt7r. The underlying data for this figure can be found in S1 Data.
(TIF)

S7 Fig GDF15 could cross the placental barrier in vitro. **(A)** Schematic of an in vitro BeWo cells transwell assay mimicking the placental barrier. rmGDF15 or PBS was added to the apical chamber of Transwell inserts with monolayer BeWo cells. FITC-dextran was then added, and the chambers were incubated for 6 h. Supernatants from the basolateral chambers were collected for further analysis. **(B)** Fluorescence intensity of FITC-dextran in the supernatant of the basolateral chamber of the Transwell with or without monolayer BeWo trophoblast cells (*n* = 6). **(C)** GDF15 levels in the supernatant of the basolateral chamber of the Transwell were measured by ELISA (*n* = 3). Data are presented as the mean ± SEM. *P* values were analyzed by two-tailed unpaired Student *t* test. *P < 0.05, **P < 0.01. rmGDF15, recombinant mouse GDF15. Panel A created in BioRender. liu, X. (2026) https://BioRender.com/ax7lt7r. The underlying data for this figure can be found in S1 Data.
(TIF)

S8 Fig Placental GDF15 could circulate into fetal liver. **(A)** Schematic diagram of mice treatment. 8-week-old pregnant dams were intraplacentally injected with Cy5.5-rGDF15 or free Cy5.5 at E17. **(B)** Fluorescence imaging of placentas (left) and fetuses (right) 3 h after intraplacental injection of Cy5.5-rGDF15 or free Cy5.5 in vivo. **(C)** Fluorescence imaging of dissected fetal organs following Cy5.5-rGDF15 administration in pregnant dam. The left panel displays fluorescence imaging of fetal organs, and the right panel displays the images of fetal organ. Radiance efficiency scales reflect photon emission intensity ((photons s^−1^ cm^−2^ sr^−1^ μW^−1^ cm^2^). Cy5.5-rGDF15, Cy5.5-labeled recombinant GDF15. Panel A created in BioRender. liu, X. (2026) https://BioRender.com/ax7lt7r.
(TIF)

S9 Fig Placenta-specific GDF15 deficiency does not alter IGF2 expression in other fetal tissues. **(A)** qPCR analysis of *Igf2* mRNA expression in various tissues of fetuses at E18.5 (*n* = 6). **(B–D)** Relative *Igf2* mRNA expression in fetal muscle (B), lung (C), and heart (D) from *Gdf15*^fl/fl^ and *Gdf15*^PKO^ fetuses (*n* = 6). **(E)** Representative immunofluorescence staining of IGF2 (Green) in muscle, lung, heart, pancreas, intestine, and brain from *Gdf15*^fl/fl^ and *Gdf15*^PKO^ fetuses. Scale bar, 200 μm. **(F and G)** western blot analysis of IGF2 protein expression in muscle (F) and lung (G) from *Gdf15*^fl/fl^ and *Gdf15*^PKO^ fetuses (*n* = 3). Data are presented as the mean ± SEM. *P* values were analyzed by two-tailed unpaired Student *t* test. *P < 0.05, **P < 0.01. The underlying data for this figure can be found in S1 Data. The Original blot for this figure can be found in S1 Raw Images.
(TIF)

S10 Fig The correlation between fetal IGF2 and fetal GDF15, placental GDF15, and fetal weight. **(A)** Pearson’s correlation coefficient analysis of plasma IGF2 and GDF15 levels in fetal mice at E18.5 (*n* = 20). **(B)** Pearson’s correlation coefficient analysis of fetal plasma IGF2 and placental GDF15 levels in pregnant mice at E18.5 (*n* = 20). **(C)** Pearson’s correlation coefficient analysis of fetal plasma IGF2 and body weight in pregnant mice at E18.5 (*n* = 20). The underlying data for this figure can be found in S1 Data.
(TIF)

S11 Fig Placental GDF15 deficiency has no effect on the STAT3 signaling pathway in fetal other tissues. **(A–D)** Relative protein expression of phosphorylated STAT3 (Tyr705) and total STAT3 in muscle (A), lung (B), heart (C), and kidney (D) from *Gdf15*^fl/fl^ and *Gdf15*^PKO^ fetuses (*n* = 3). The Original blot for this figure can be found in S1 Raw Images.
(TIF)

S12 Fig STAT3 binds with the promoter of murine *Igf2.* **(A)** Schematic images of structure and putative STAT3-binding motif in the P3 of murine *Igf2* gene predicted by JASPAR. **(B)** Wild-type and mutant *Igf2* P3 promoter luciferase reporter. **(C)** Luciferase activity of wild-type or mutant *Igf2* reporters in the presence of STAT3 (*n* = 6). **(D)** Luciferase reporter analysis in WT or *Stat3*-OE primary mouse fetal hepatocytes transfected with *Igf2* P3 constructs (*n* = 6). **(E)** ChIP analysis of STAT3 occupancy in the *Igf2* P3 in primary mouse fetal hepatocytes (*n* = 3). Data are presented as the mean ± SEM. *P* values were analyzed by two-tailed unpaired Student *t* test. *P < 0.05, **P < 0.01. E, exon; P, promoter. The underlying data for this figure can be found in S1 Data.
(TIF)

S1 Table Genotype identification primers used in this study. (DOCX)

S2 Table qRT-PCR primers used in this study. (DOCX)

S3 Table Primary antibodies used in this study. (DOCX)

S4 Table Linear mixed-effects model (LMM) analyses of fetal growth outcomes. (DOCX)

S1 Data Contains underlying data for Figs 1A, 1B, 1D–1I, 1K–1N, 2B, 2C, 2F–2K, 3E, 3F, 3I–3L, 4B, 4C, 4G–4M, 5B, 5C, 5E, 5F, 5I, 6A, 6D–6F, 6H, 7A, 7D, 7G–7I, S1A–S1C, S1F–S1P, S2D, S2F–S2I, S3A, S4A, S5A–S5F, S6A–S6C, S6G–S6N, S7B, S7C, S9A–S9D, S10A–S10C, S12C–S12E. (ZIP)

S1 Raw Images Uncropped version of all western blotting images in the main body and supporting information. (PDF)

## Abbreviations

ALT: alanine transaminase
DLS: Dynamic light scattering
ELISA: Enzyme-linked immunosorbent assay
FC: fold change
FGR: Fetal growth restriction
GH: growth hormone
IGF: insulin-like growth factor
KEGG: Kyoto Encyclopedia of Genes and Genomes
LMM: Linear mixed-effects model
LNP: lipid nanoparticle
MS: Mass spectrometry
P2: promoters 2
PDI: polydispersity index
STAT3: Signal Transducer and Activator of Transcription 3
WT: wild-type
