# Supplementary material for: Deficiency of placental GDF15 causes fetal growth restriction by inhibiting fetal hepatic STAT3-IGF2 axis
Source: PLoS Biol. 2026 Oct 14;24(10):e3004061. doi: 10.1371/journal.pbio.3004061 (PMC13686457; doi:10.1371/journal.pbio.3004061)
Supplement: Supplementary file 1 [file pbio.3004061.s018.pdf]

Full and uncropped western blots

Western blots (WB) and raw data are shown in the following pattern

Up： Western blots shown in Figures

Bottom : Full and uncropped Western blots images merged with membrane with molecular weight markers

Fig 3B

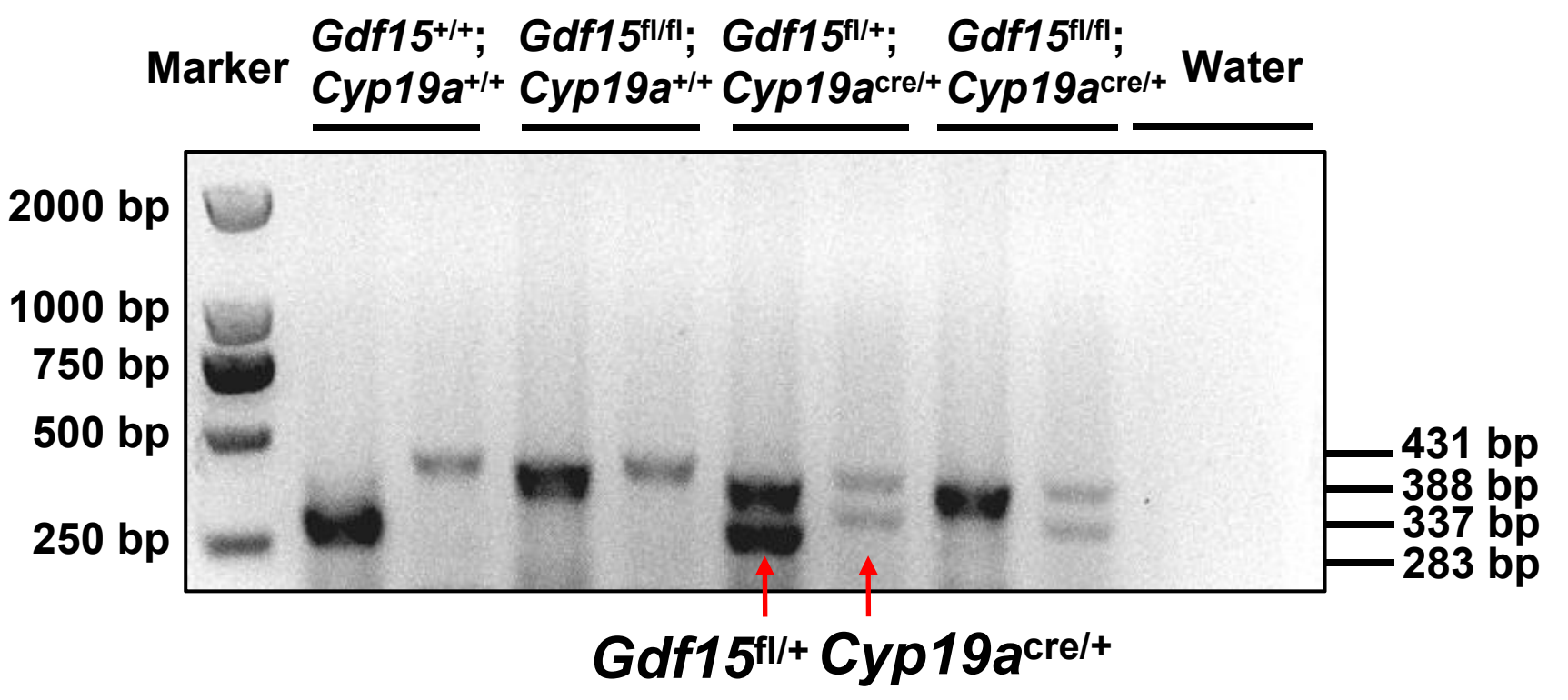

Marker
Gdf15+/+; Cyp19a+/+
Gdf15fl/fl; Cyp19a+/+
Gdf15fl/+; Cyp19acre/+
Gdf15fl/fl; Cyp19acre/+
Water
2000 bp
1000 bp
750 bp
500 bp
250 bp
431 bp
388 bp
337 bp
283 bp
Gdf15fl/+
Cyp19acre/+

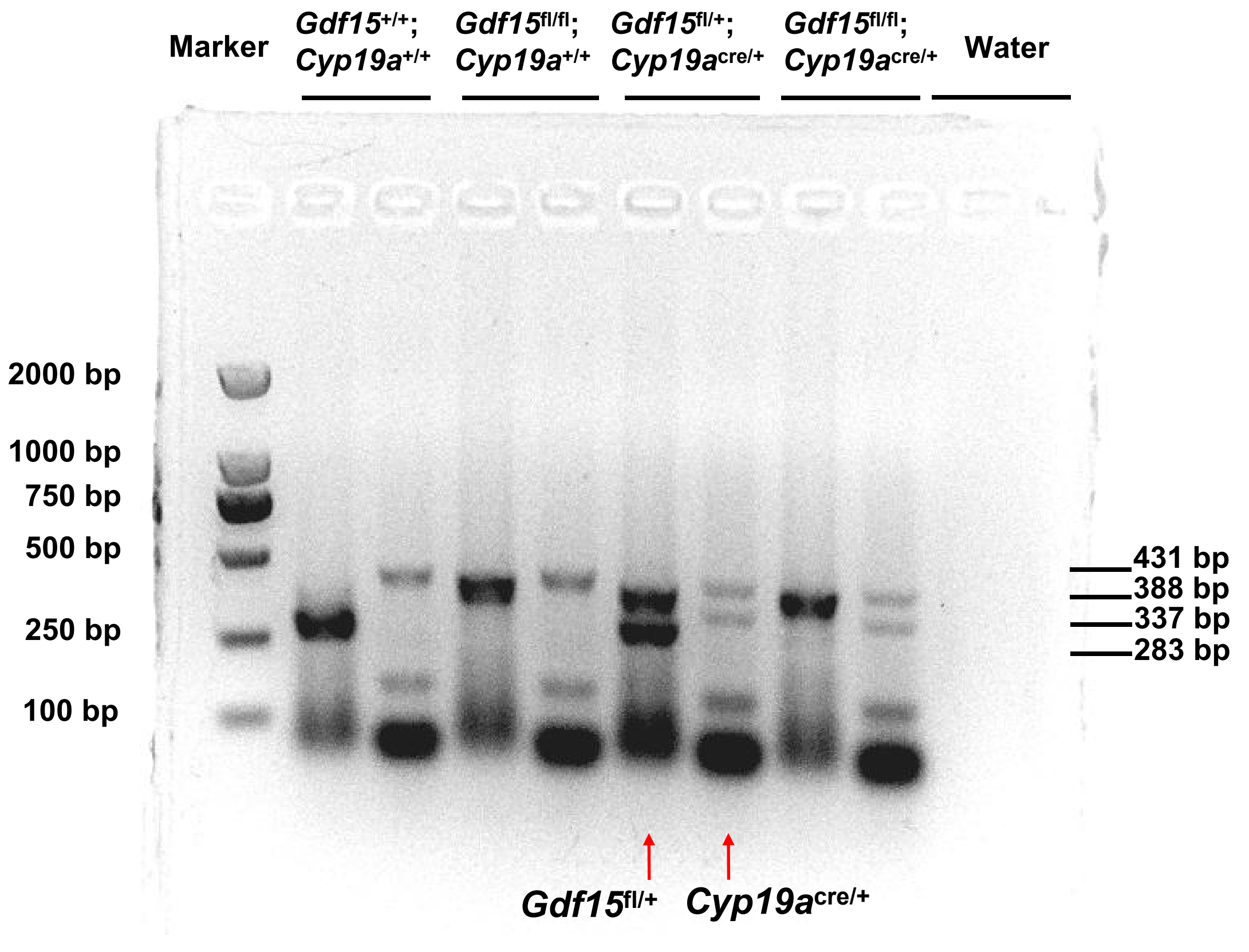

Marker
Gdf15+/+; Cyp19a+/+
Gdf15fl/fl; Cyp19a+/+
Gdf15fl/+; Cyp19acre/+
Gdf15fl/fl; Cyp19acre/+
Water
2000 bp
1000 bp
750 bp
500 bp
250 bp
100 bp
431 bp
388 bp
337 bp
283 bp
Gdf15fl/+
Cyp19acre/+

Fig 5G

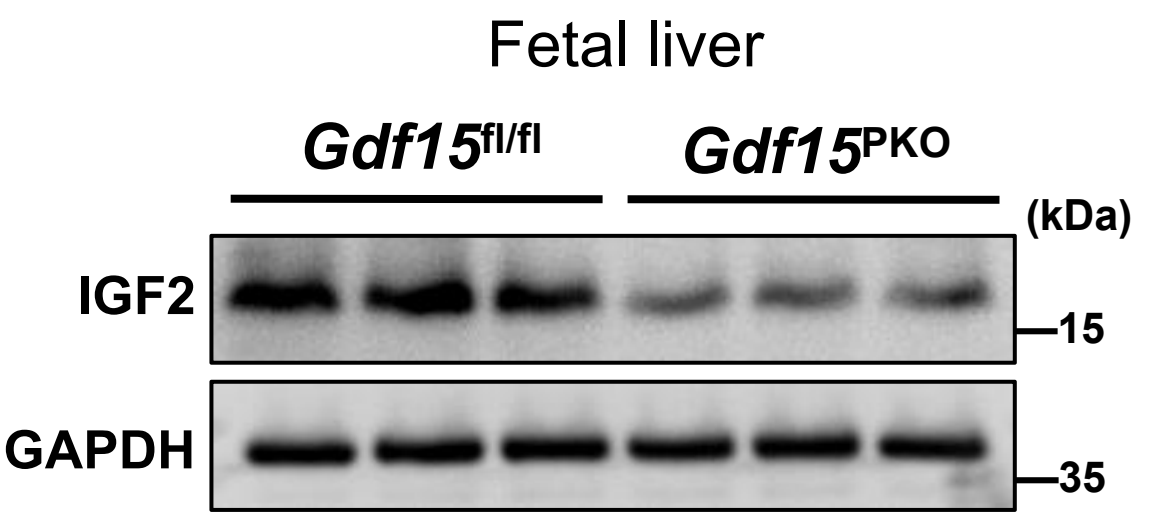
Fetal liver
Gdf15fl/fl
Gdf15PKO
(kDa)
IGF2
15
GAPDH
35

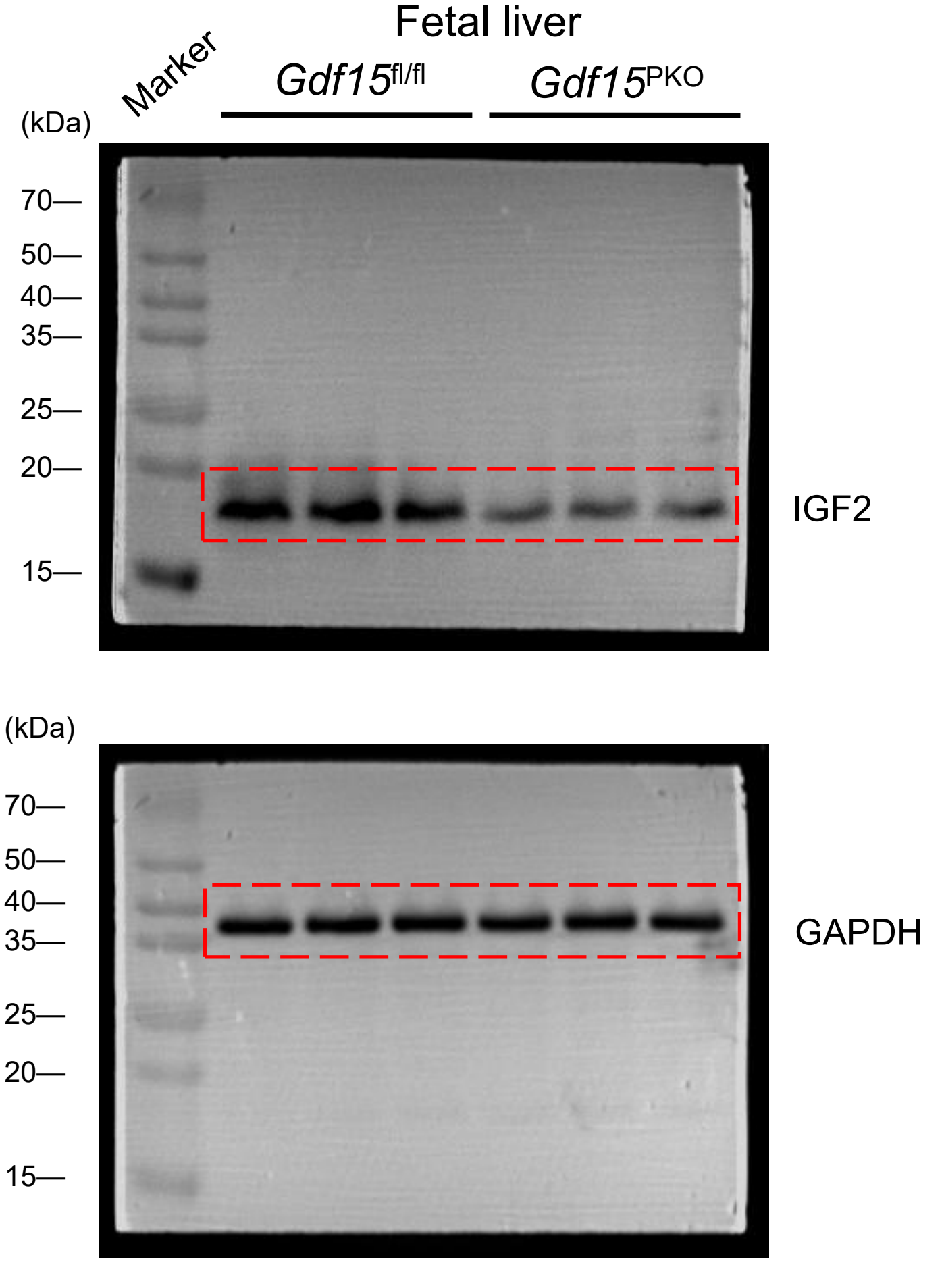
Fetal liver
Marker
Gdf15fl/fl
Gdf15PKO
(kDa)
70
50
40
35
25
20
15
IGF2
(kDa)
70
50
40
35
25
20
15
GAPDH

Fig 6B

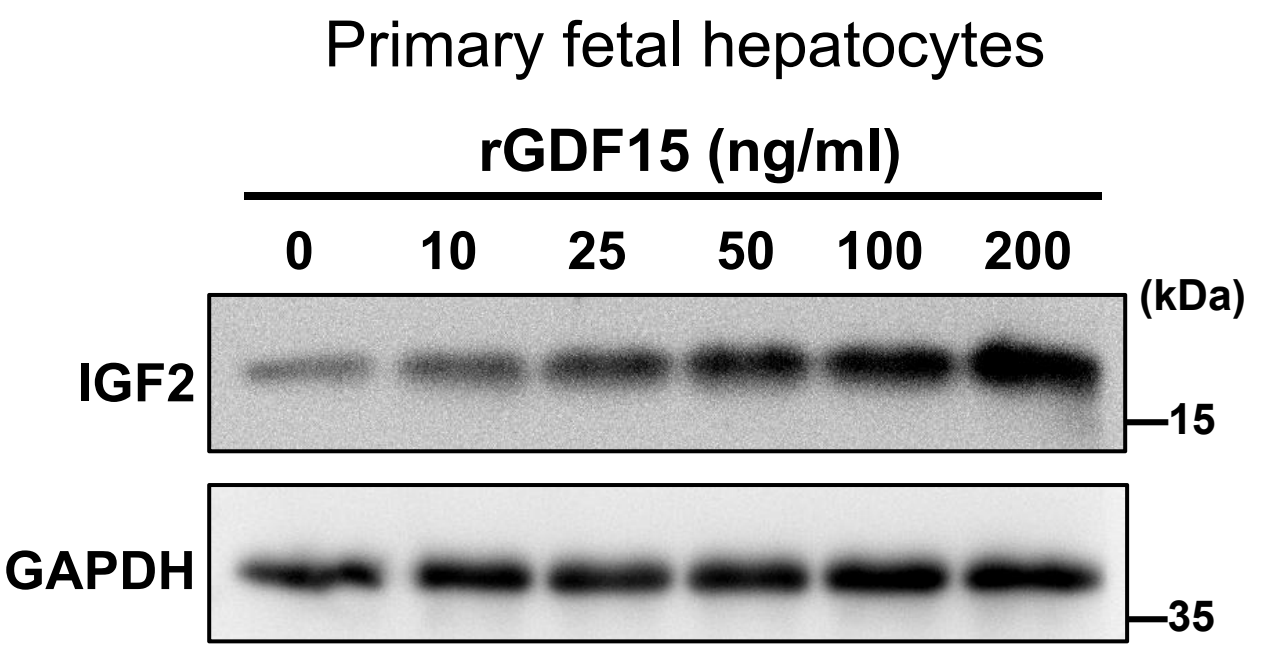
Primary fetal hepatocytes
rGDF15 (ng/ml)
0 10 25 50 100 200
(kDa)
IGF2
15
GAPDH
35

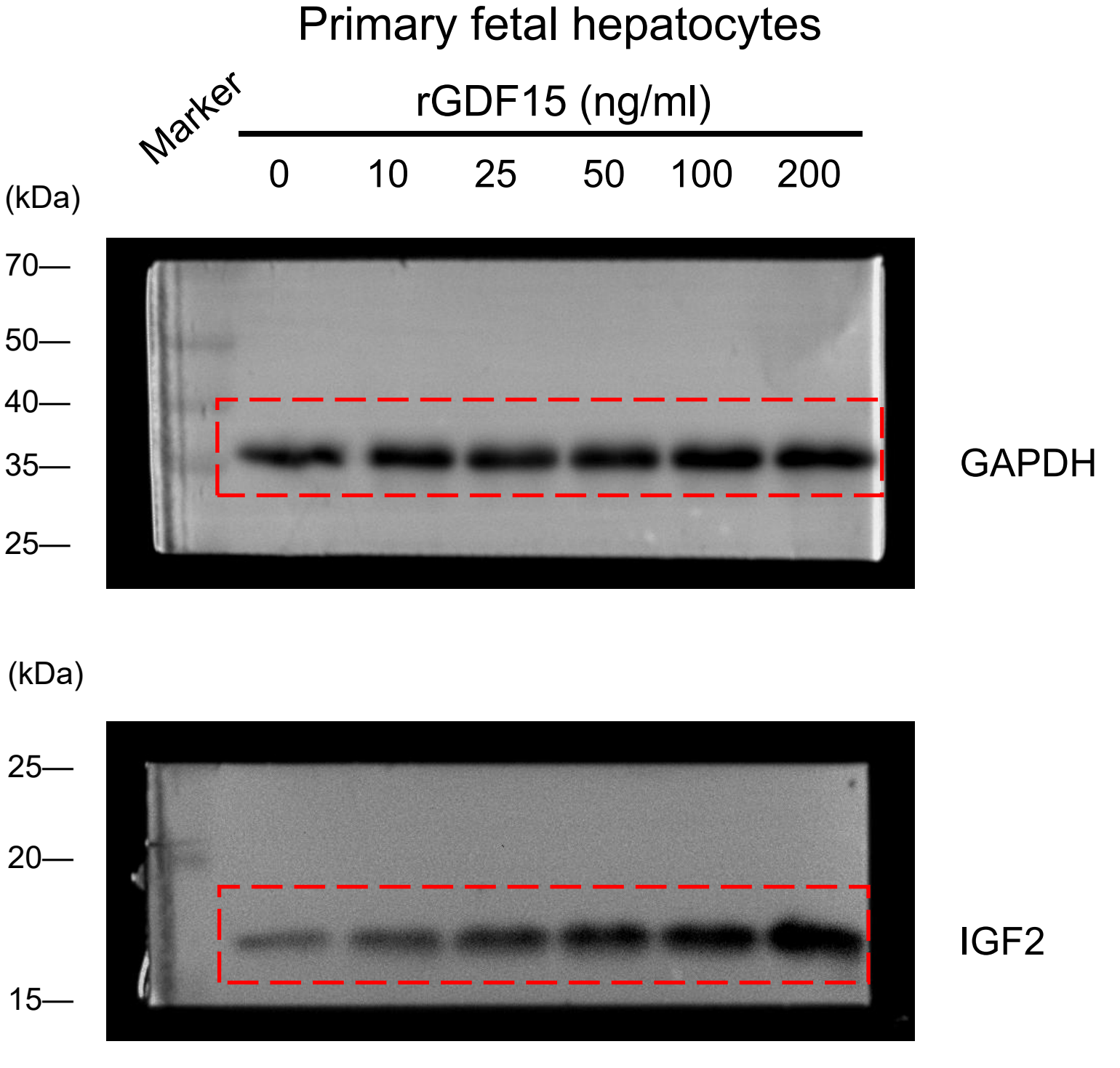
Primary fetal hepatocytes
Marker
rGDF15 (ng/ml)
0 10 25 50 100 200
(kDa)
70
50
40
35
25
GAPDH
(kDa)
25
20
15
IGF2

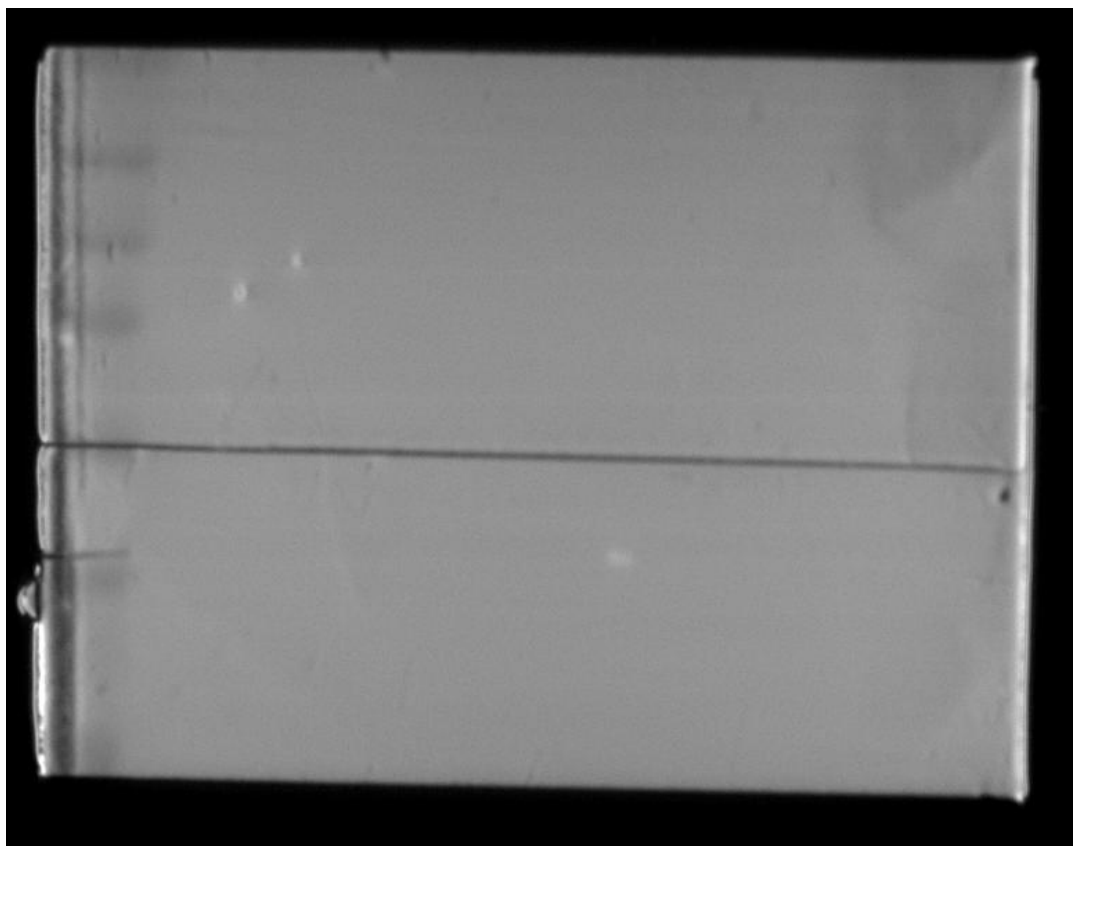

Fig 6G

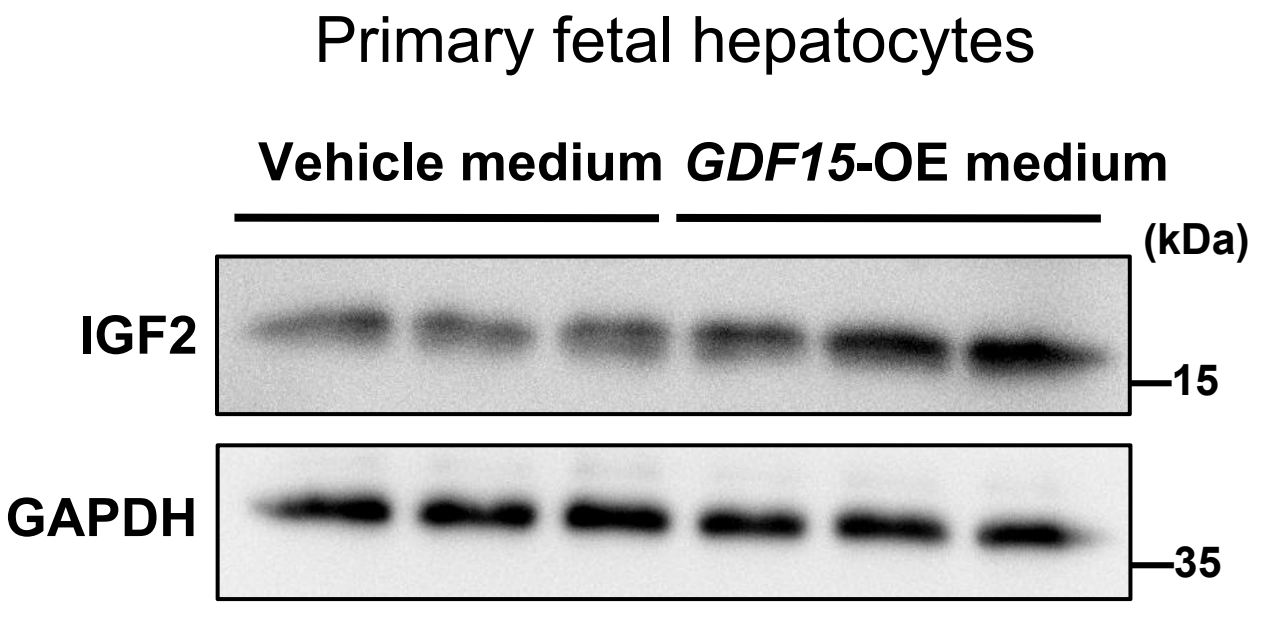
Primary fetal hepatocytes
Vehicle medium
GDF15-OE medium
(kDa)
IGF2
15
GAPDH
35

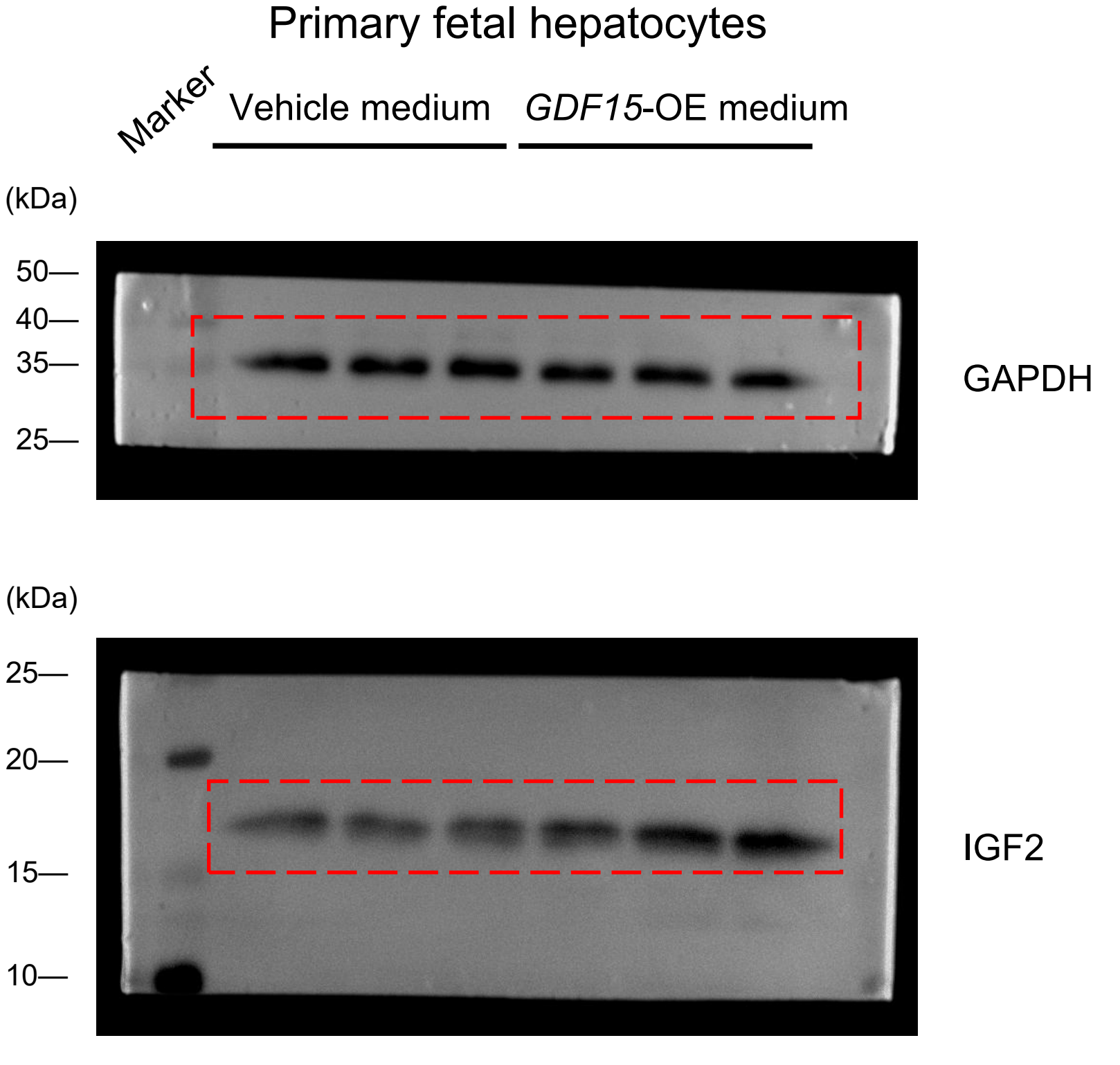
Primary fetal hepatocytes
Marker
Vehicle medium
GDF15-OE medium
(kDa)
50
40
35
25
GAPDH
(kDa)
25
20
15
10
IGF2

Fig 7B

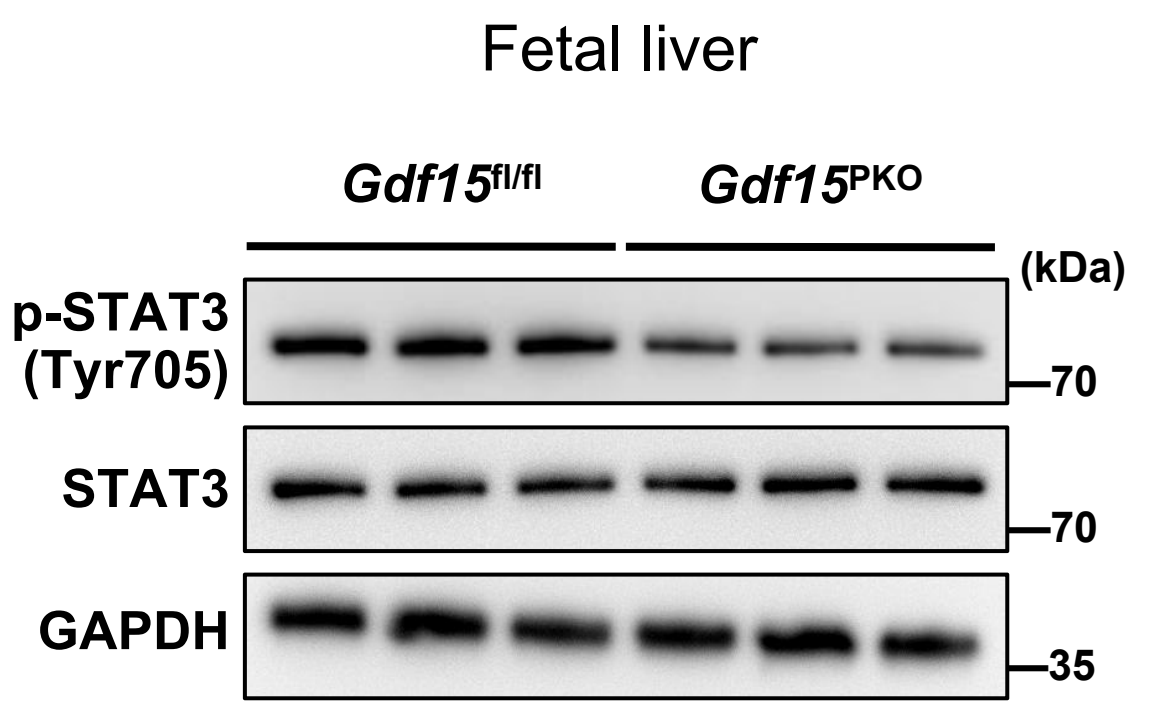

Fetal liver
Gdf15fl/fl
Gdf15PKO
(kDa)
p-STAT3
(Tyr705)
70
STAT3
70
GAPDH
35

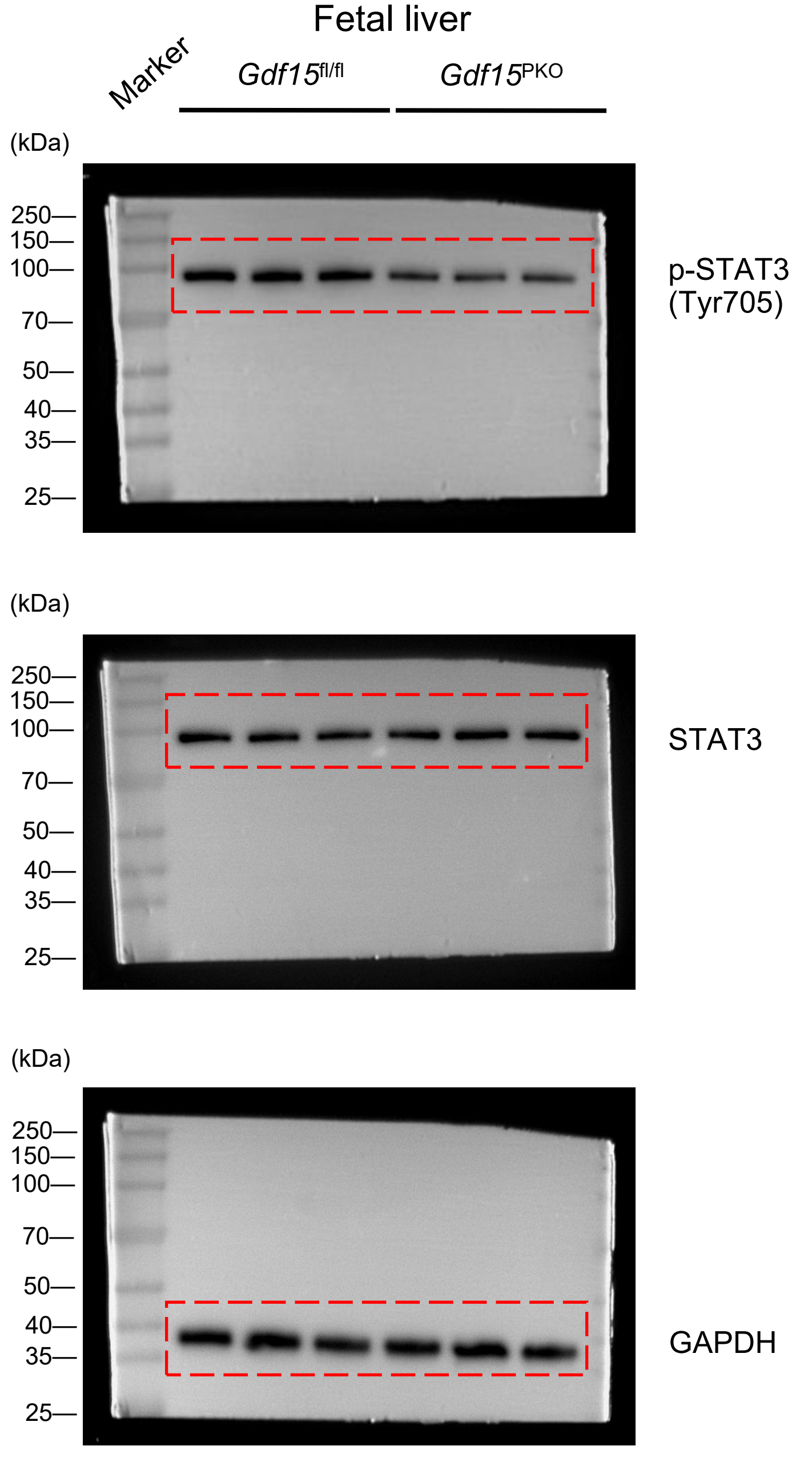

Fetal liver
Marker
Gdf15fl/fl
Gdf15PKO
(kDa)
250
150
100
70
50
40
35
25
p-STAT3
(Tyr705)
(kDa)
250
150
100
70
50
40
35
25
STAT3
(kDa)
250
150
100
70
50
40
35
25
GAPDH

Fig 7C

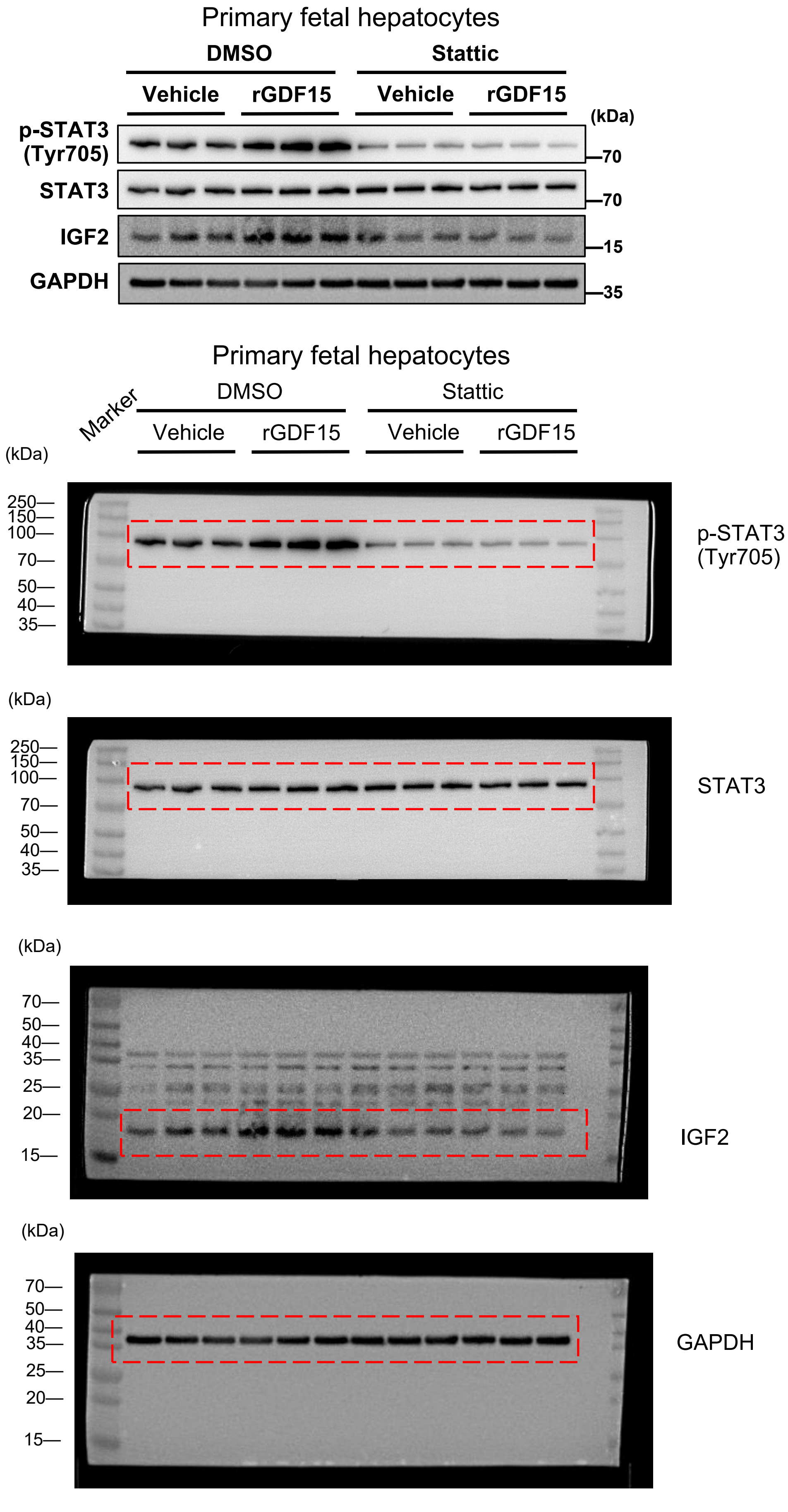

Primary fetal hepatocytes
DMSO
Stattic
Vehicle
rGDF15
Vehicle
rGDF15
(kDa)
p-STAT3 (Tyr705)
STAT3
IGF2
GAPDH
70
70
15
35
Primary fetal hepatocytes
Marker
DMSO
Stattic
Vehicle
rGDF15
Vehicle
rGDF15
(kDa)
250—
150—
100—
70—
50—
40—
35—
p-STAT3 (Tyr705)
(kDa)
250—
150—
100—
70—
50—
40—
35—
STAT3
(kDa)
70—
50—
40—
35—
25—
20—
15—
IGF2
(kDa)
70—
50—
40—
35—
25—
20—
15—
GAPDH

Fig 7E

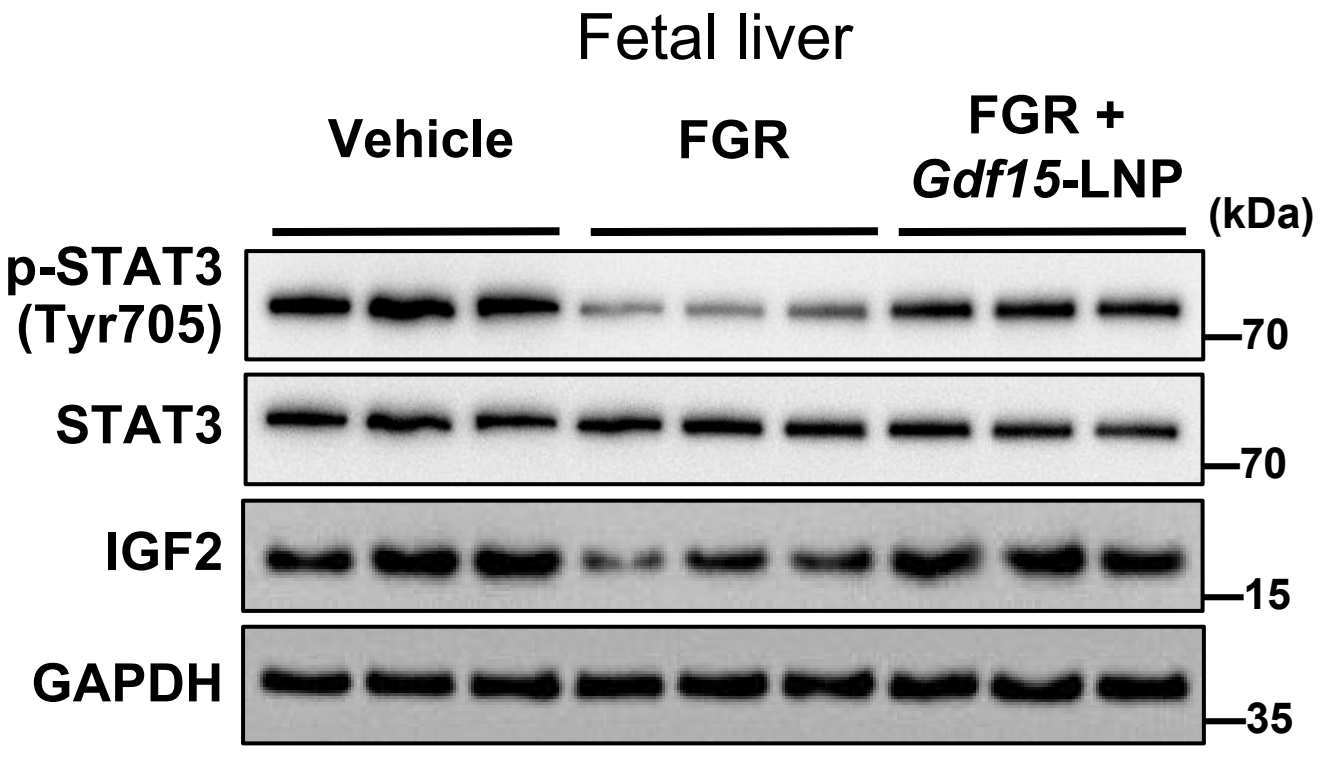

Fetal liver
Vehicle
FGR
FGR + Gdf15-LNP
(kDa)
p-STAT3 (Tyr705)
STAT3
IGF2
GAPDH
70
70
15
35

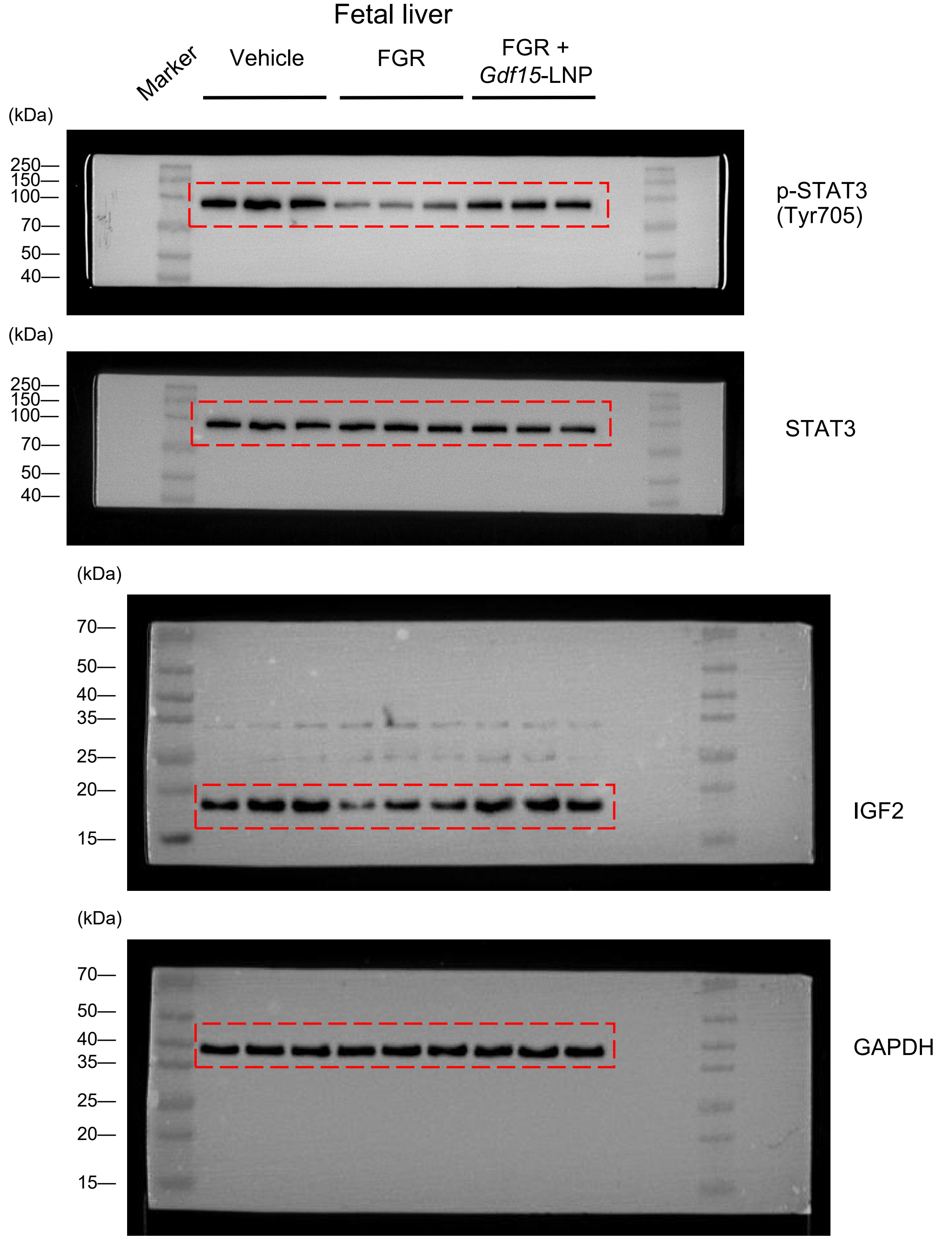

Fetal liver
Marker
Vehicle
FGR
FGR + Gdf15-LNP
(kDa)
250
150
100
70
50
40
p-STAT3 (Tyr705)
(kDa)
250
150
100
70
50
40
STAT3
(kDa)
70
50
40
35
25
20
15
IGF2
(kDa)
70
50
40
35
25
20
15
GAPDH

S2B Fig

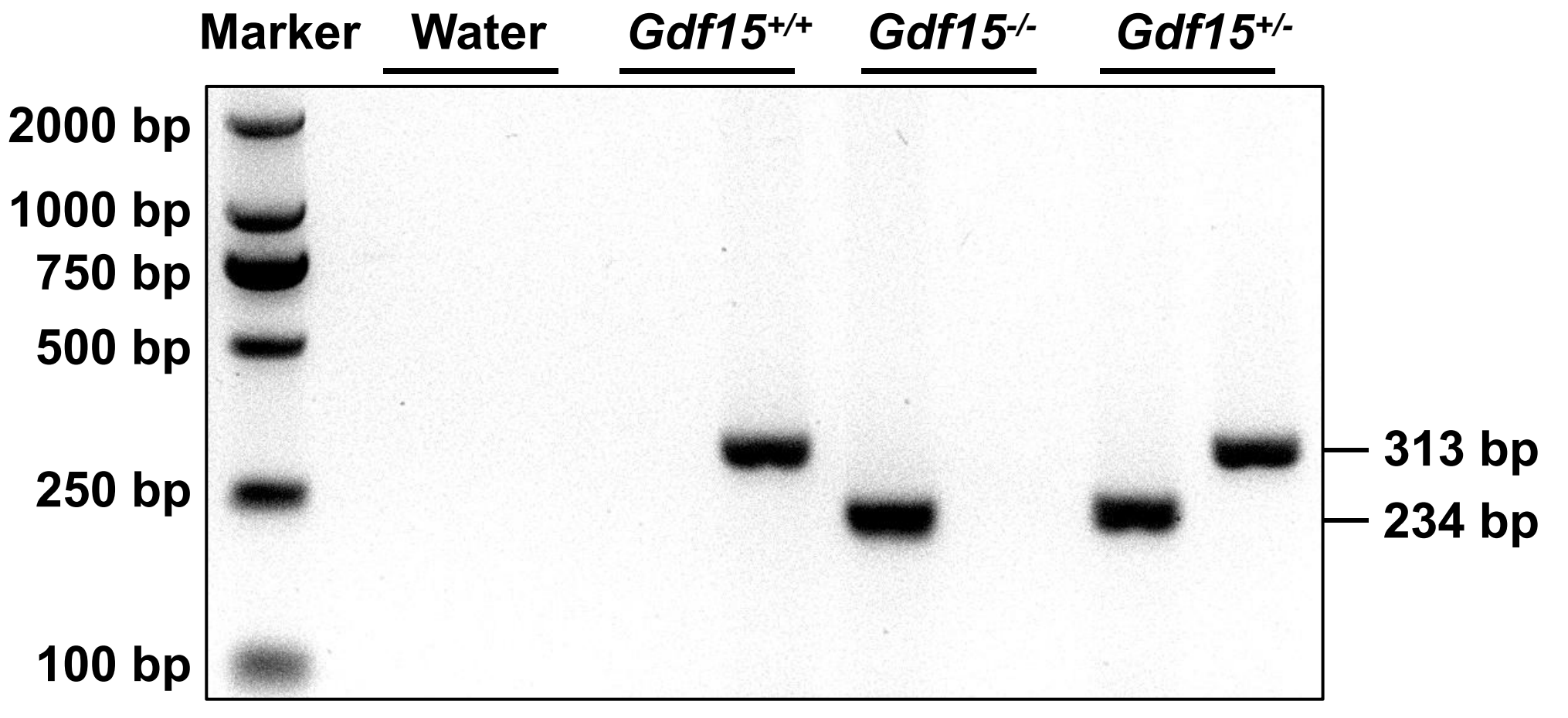
Marker
Water
Gdf15+/+
Gdf15-/-
Gdf15+/-
2000 bp
1000 bp
750 bp
500 bp
250 bp
100 bp
313 bp
234 bp

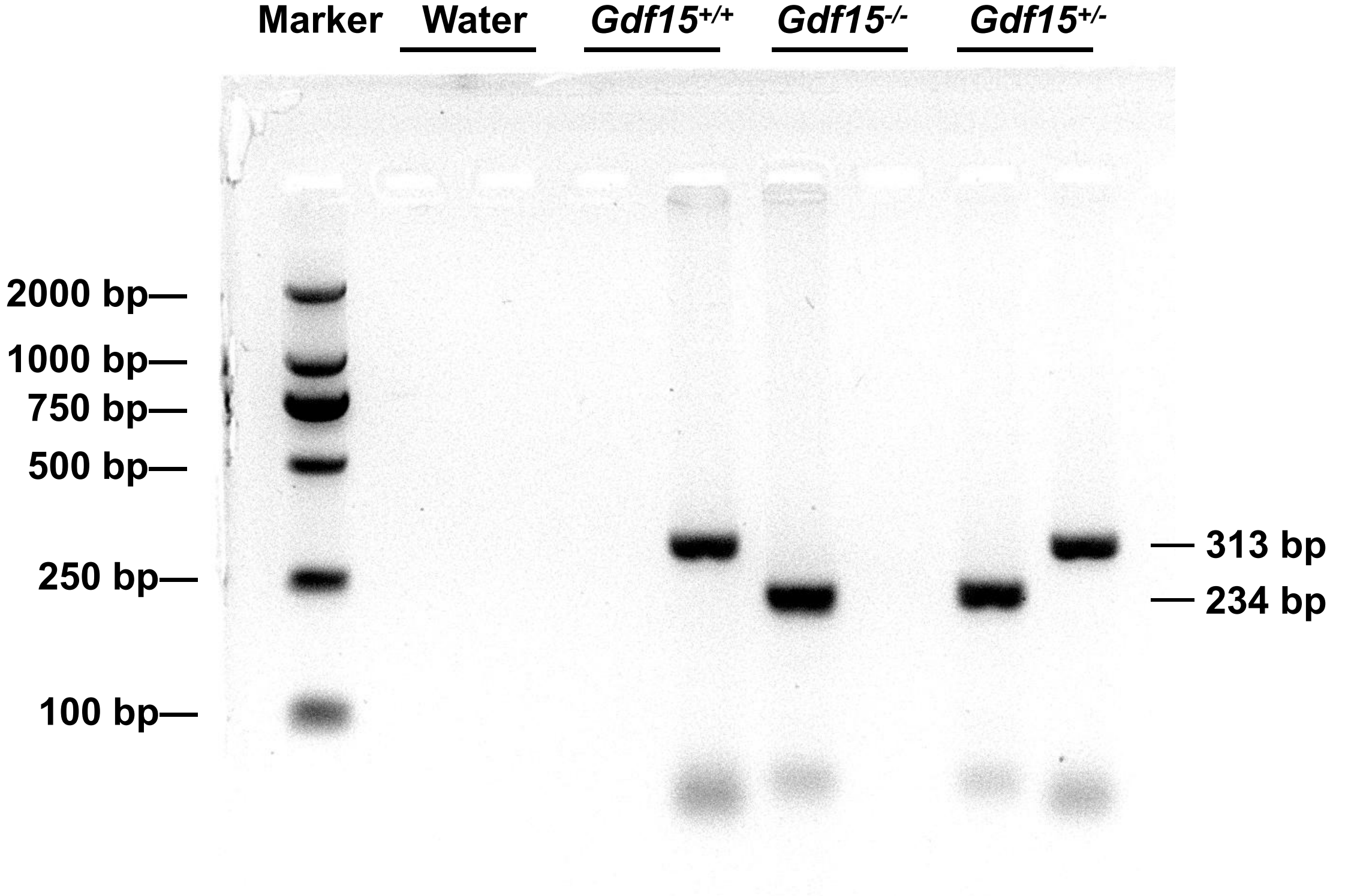
Marker
Water
Gdf15+/+
Gdf15-/-
Gdf15+/-
2000 bp
1000 bp
750 bp
500 bp
250 bp
100 bp
313 bp
234 bp

S4B Fig

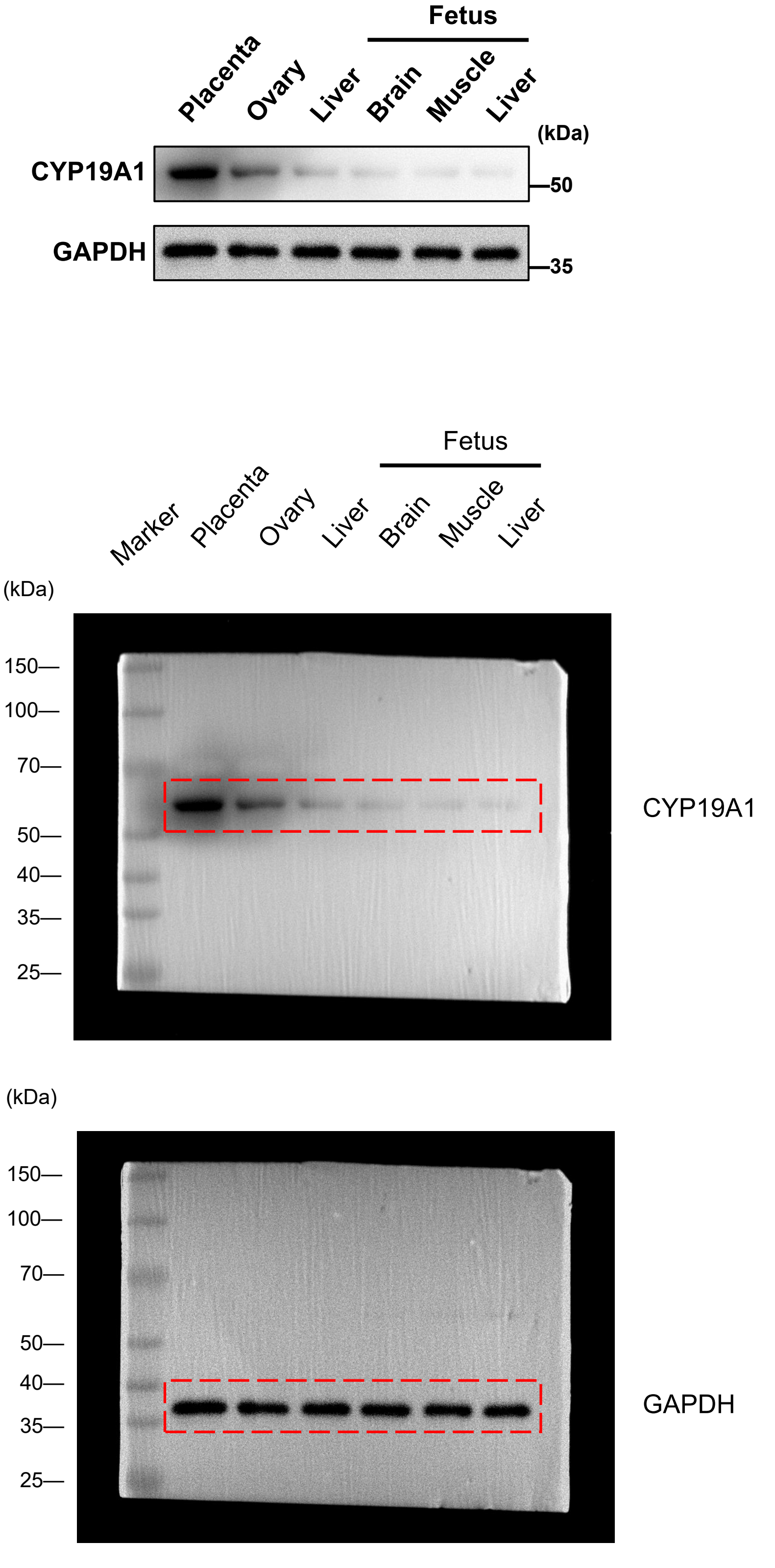
Fetus
Placenta
Ovary
Liver
Brain
Muscle
Liver
(kDa)
CYP19A1
50
GAPDH
35
Fetus
Marker
Placenta
Ovary
Liver
Brain
Muscle
Liver
(kDa)
150—
100—
70—
50—
40—
35—
25—
CYP19A1
(kDa)
150—
100—
70—
50—
40—
35—
25—
GAPDH

S9F Fig

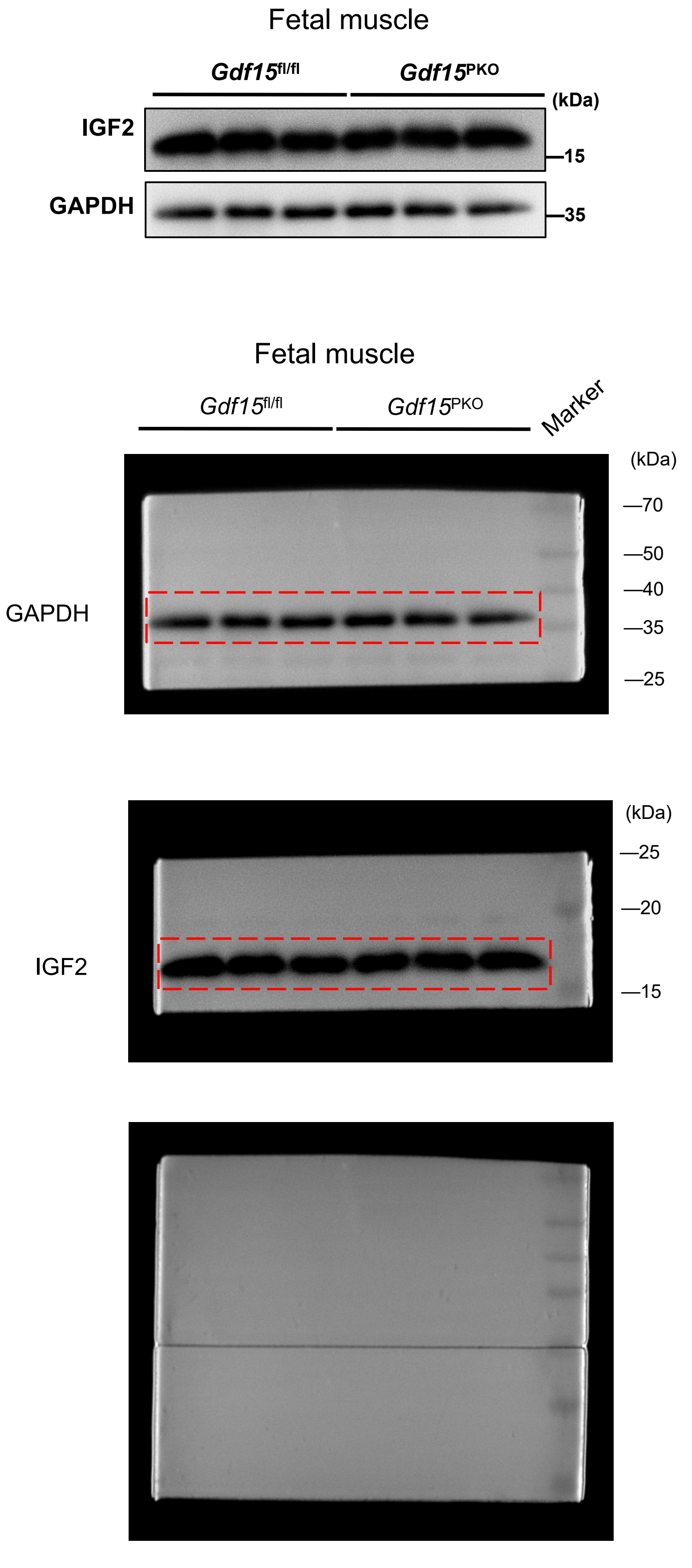

Fetal muscle
Gdf15fl/fl
Gdf15PKO
(kDa)
IGF2
15
GAPDH
35
Fetal muscle
Gdf15fl/fl
Gdf15PKO
Marker
(kDa)
70
50
40
35
25
GAPDH
(kDa)
25
20
15
IGF2

S9G Fig

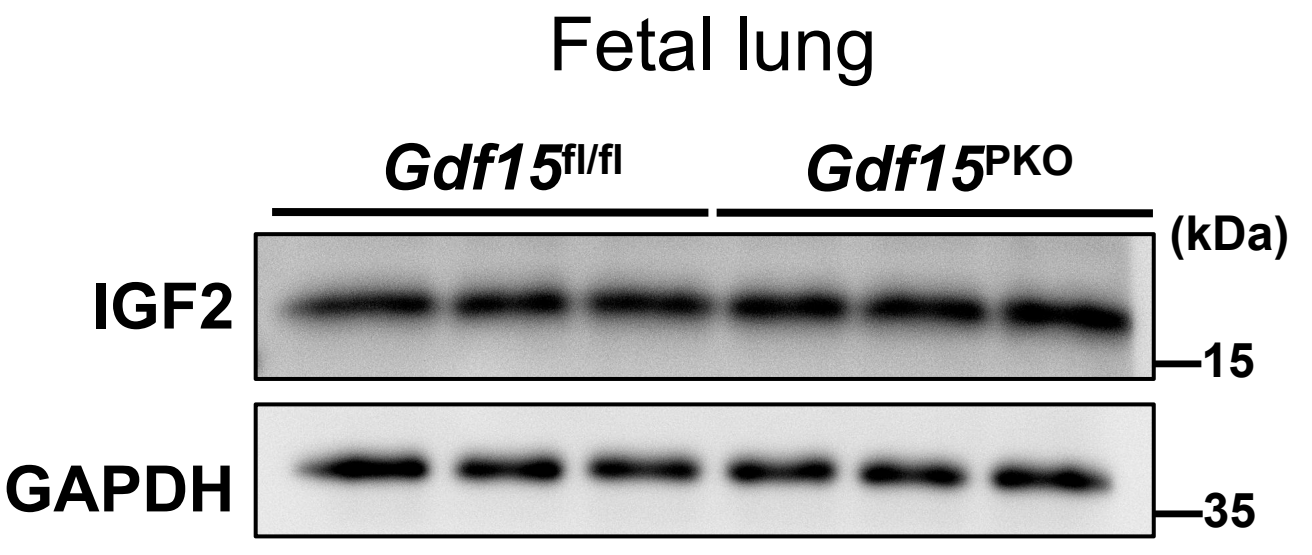
Fetal lung
Gdf15fl/fl
Gdf15PKO
(kDa)
IGF2
15
GAPDH
35

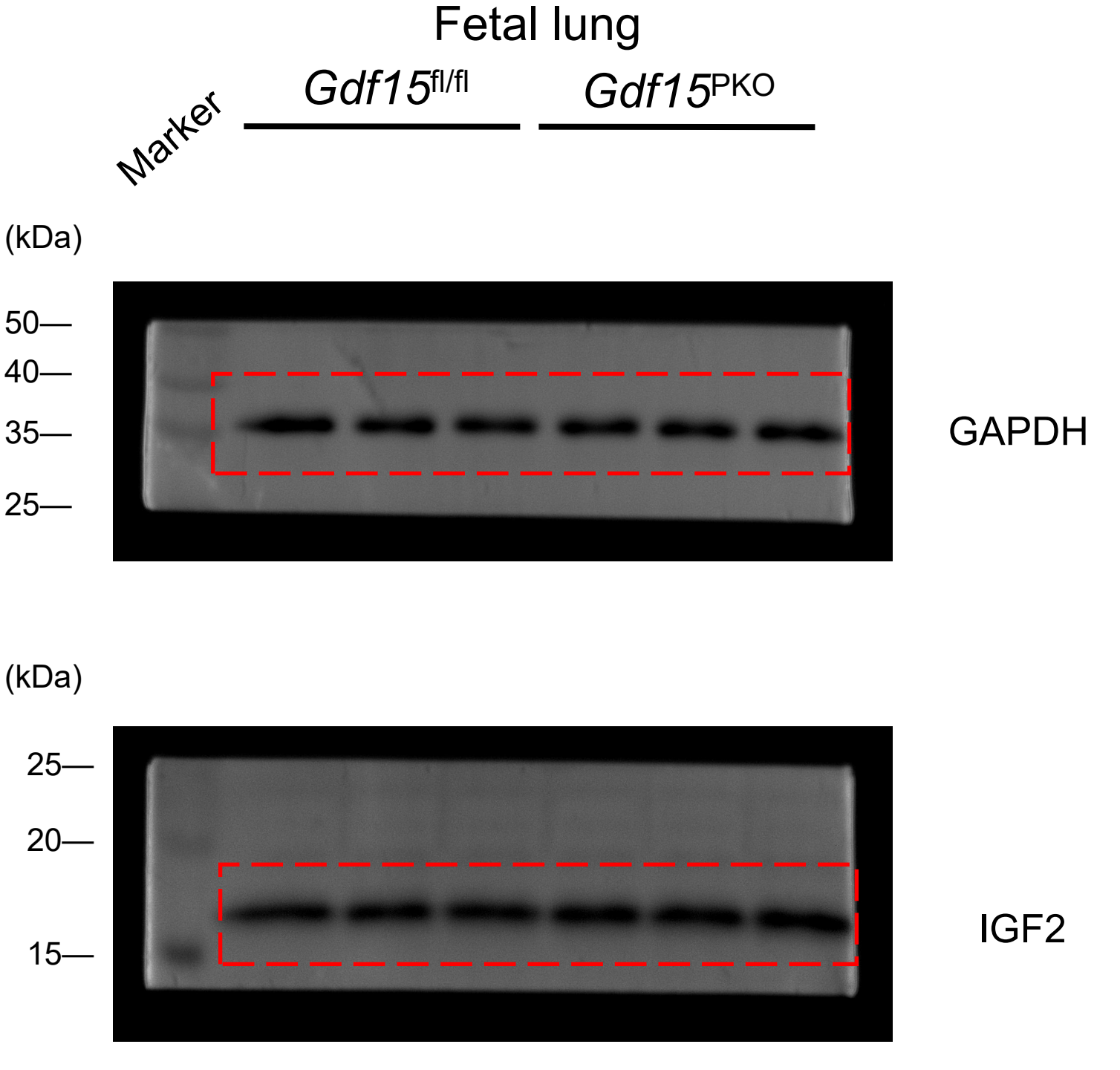
Fetal lung
Marker
Gdf15fl/fl
Gdf15PKO
(kDa)
50—
40—
35—
25—
GAPDH
(kDa)
25—
20—
15—
IGF2

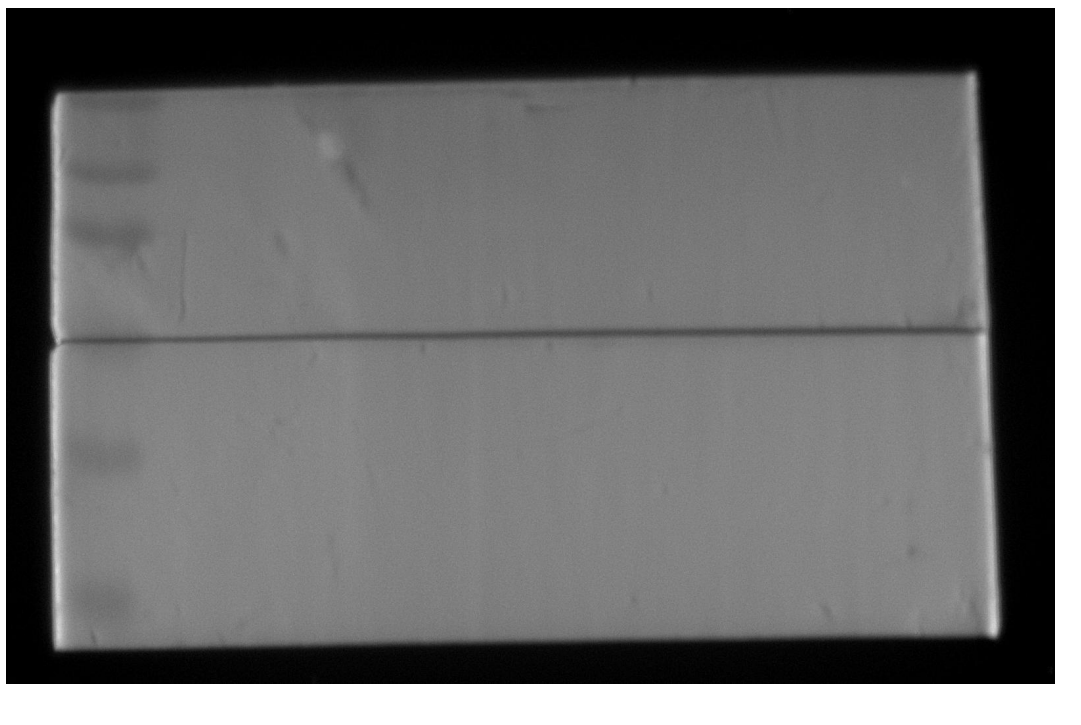

S11A Fig

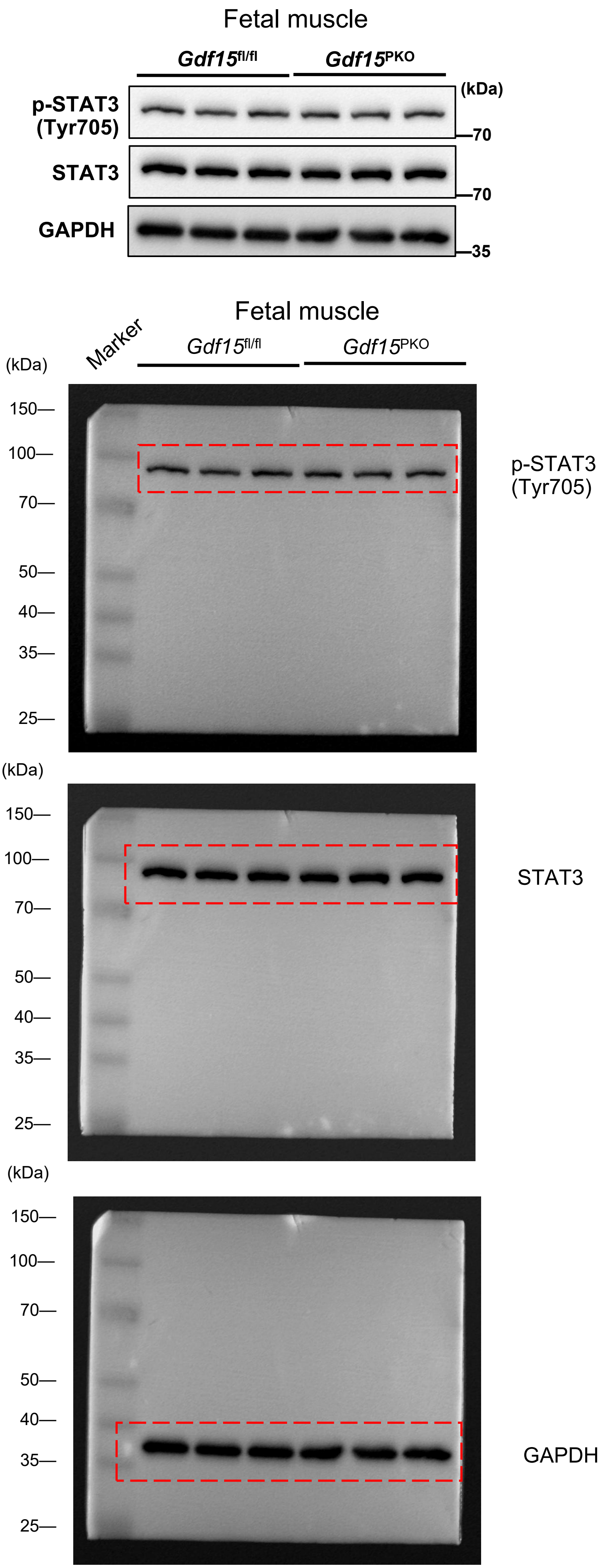

Fetal muscle
Gdf15fl/fl
Gdf15PKO
p-STAT3 (Tyr705)
STAT3
GAPDH
(kDa)
70
70
35
Fetal muscle
Marker
Gdf15fl/fl
Gdf15PKO
(kDa)
150
100
70
50
40
35
25
p-STAT3 (Tyr705)
(kDa)
150
100
70
50
40
35
25
STAT3
(kDa)
150
100
70
50
40
35
25
GAPDH

S11B Fig

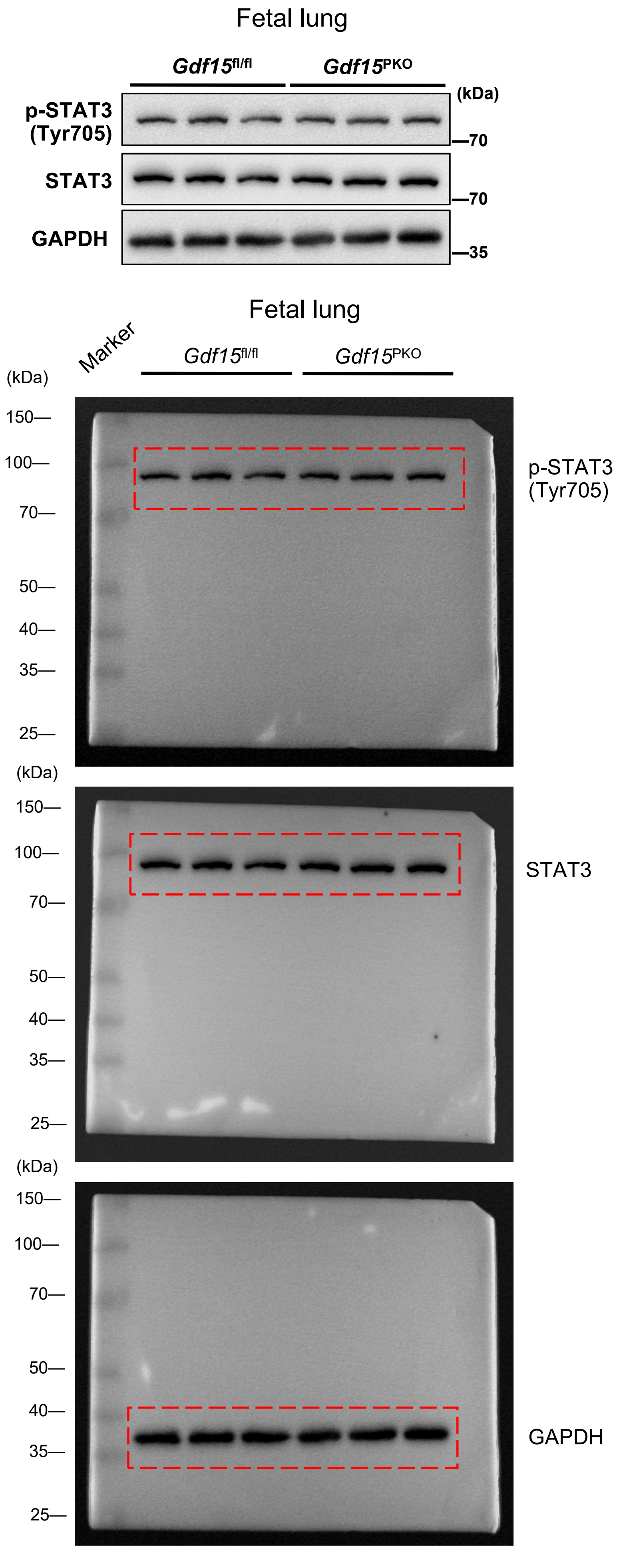

Fetal lung
Gdf15fl/fl
Gdf15PKO
(kDa)
p-STAT3 (Tyr705)
STAT3
GAPDH
—70
—70
—35
Fetal lung
Marker
Gdf15fl/fl
Gdf15PKO
(kDa)
150—
100—
70—
50—
40—
35—
25—
p-STAT3 (Tyr705)
STAT3
GAPDH

S11C Fig

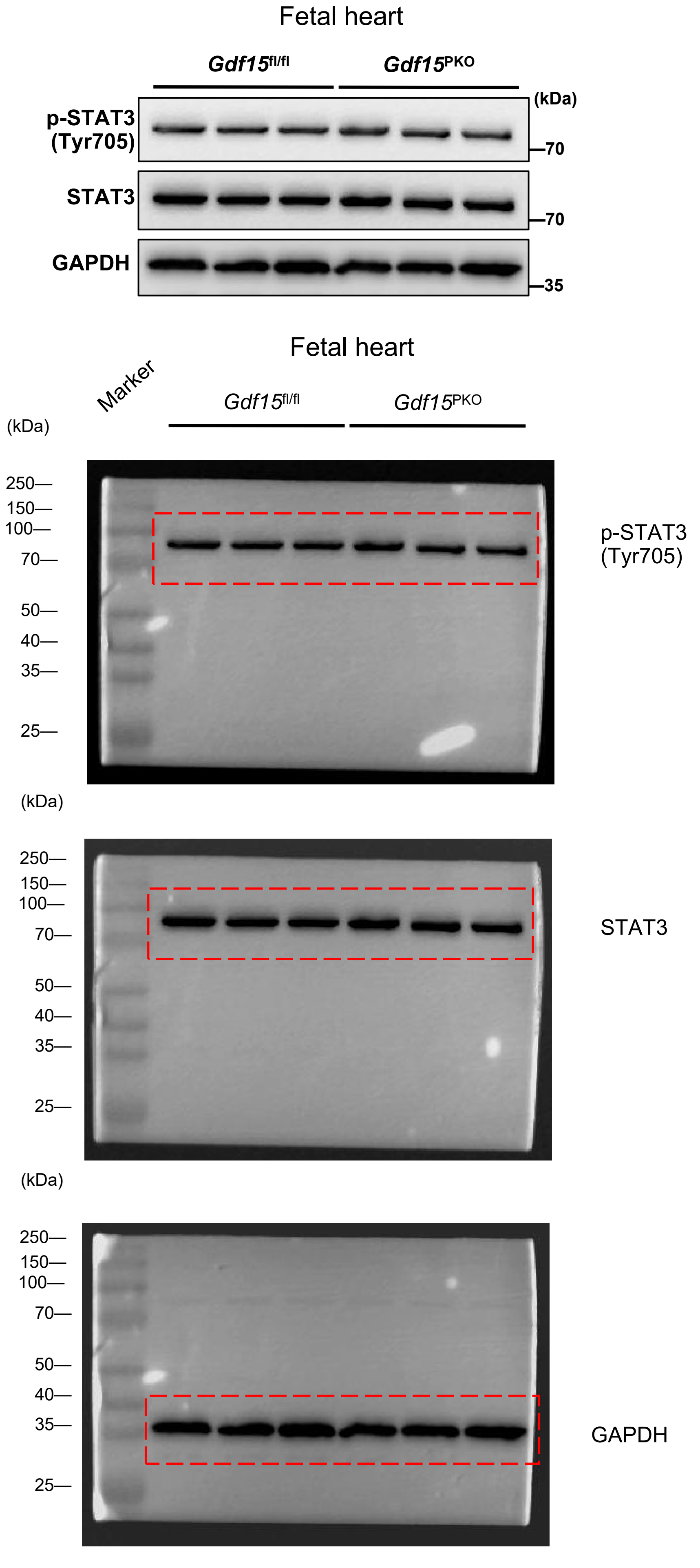

Fetal heart
Gdf15fl/fl
Gdf15PKO
(kDa)
p-STAT3 (Tyr705)
—70
STAT3
—70
GAPDH
—35
Fetal heart
Marker
Gdf15fl/fl
Gdf15PKO
(kDa)
250—
150—
100—
70—
50—
40—
35—
25—
p-STAT3 (Tyr705)
(kDa)
250—
150—
100—
70—
50—
40—
35—
25—
STAT3
(kDa)
250—
150—
100—
70—
50—
40—
35—
25—
GAPDH

S11D Fig

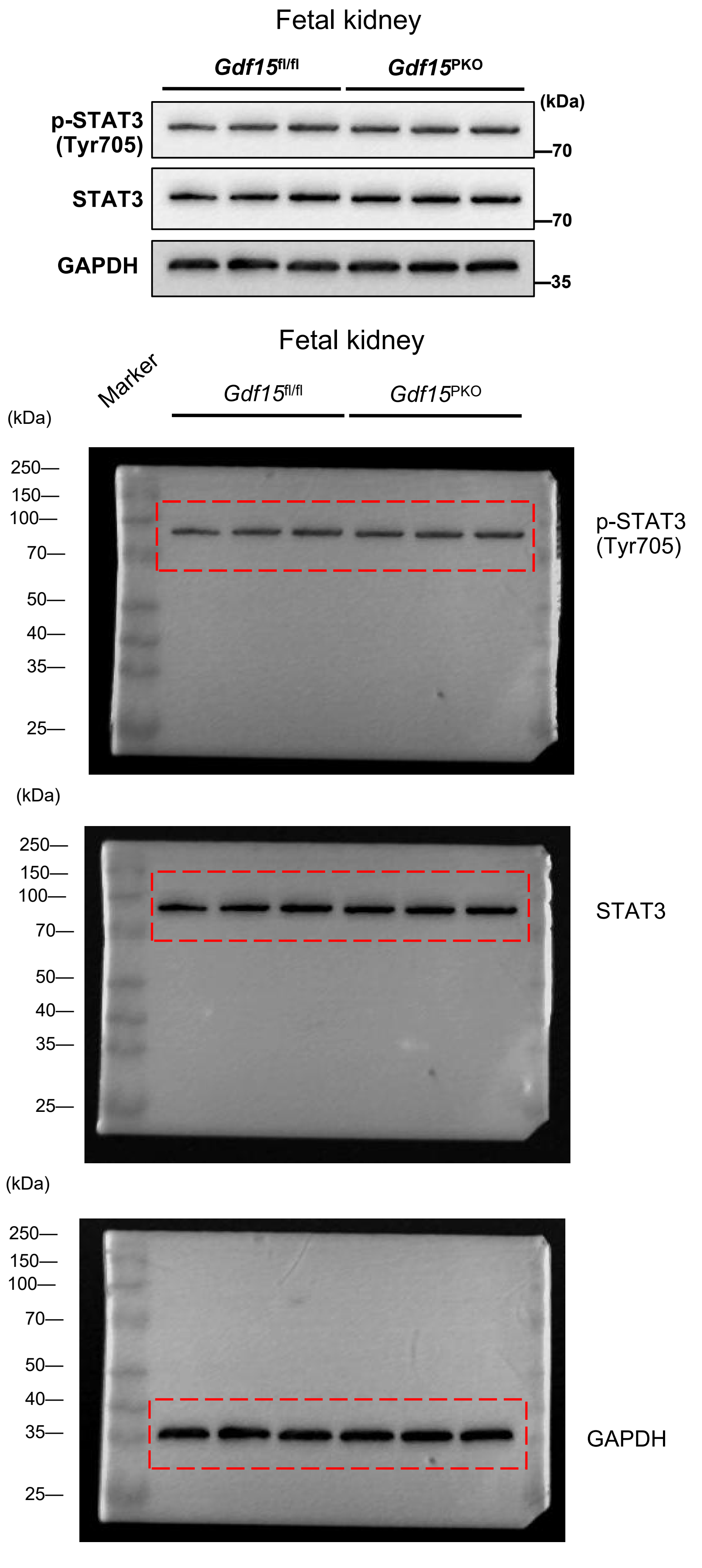
Fetal kidney
Gdf15fl/fl
Gdf15PKO
(kDa)
p-STAT3
(Tyr705)
70
STAT3
70
GAPDH
35
Fetal kidney
Marker
Gdf15fl/fl
Gdf15PKO
(kDa)
250—
150—
100—
70—
50—
40—
35—
25—
p-STAT3
(Tyr705)
(kDa)
250—
150—
100—
70—
50—
40—
35—
25—
STAT3
(kDa)
250—
150—
100—
70—
50—
40—
35—
25—
GAPDH
