# Supplementary material for: Deficiency of placental GDF15 causes fetal growth restriction by inhibiting fetal hepatic STAT3-IGF2 axis
Source: PLoS Biol. 2026 Oct 14;24(10):e3004061. doi: 10.1371/journal.pbio.3004061 (PMC13686457; doi:10.1371/journal.pbio.3004061)
Supplement: Supplementary file 2 [file pbio.3004061.s013.docx]

**S1 Table. Genotype identification primers used in this study.**

| **Genotype** | **Primers** | **Size (bp)** |
| --- | --- | --- |
| *Gdf15*^+/+^ | F: CACTTGAACTTCAGTCTGGACTCC | 313 |
|  | R: CAGCAGGAACAGCAGGAACCTCAG |  |
| *Gdf15*^−/−^ | F: CACTTGAACTTCAGTCTGGACTCC | 234 |
|  | R: CCTTGCTGCTCAAACACCCAGGAC |  |
| *Gdf15*-Flox | F: AAGAACCTGCGGGAACTGGATC | 388 |
|  | R: CCGGAGTAGTTAGGATGGCAGAC |  |
| *Cyp19a1*-Cre | F: TACACTTTTGAGACGATTCCAGGT | 337 |
|  | R: TACTGATGGTCTGTCTTTCCCAAG |  |
