# Supplementary material for: Deficiency of placental GDF15 causes fetal growth restriction by inhibiting fetal hepatic STAT3-IGF2 axis
Source: PLoS Biol. 2026 Oct 14;24(10):e3004061. doi: 10.1371/journal.pbio.3004061 (PMC13686457; doi:10.1371/journal.pbio.3004061)
Supplement: Supplementary file 3 [file pbio.3004061.s014.docx]

**S2 Table. qRT-PCR primers used in this study.**

| **Species** | **Gene** | **Primers** | **Size (bp)** | **Accession Number** |
| --- | --- | --- | --- | --- |
| Mouse | *Gapdh* | F: TGCCCAGAACATCATCCCT | 233 | NM_001289726.2 |
|  |  | R: GGTCCTCAGTGTAGCCCAAG |  |  |
|  | *Gdf15* | F: GAGCTACGGGGTCGCTTC | 130 | NM_011819.4 |
|  |  | R: GGGACCCCAATCTCACCT |  |  |
|  | *Igf2* | F: CGGCTTCTACTTCAGCAGGC | 163 | NM_001122737.2 |
|  |  | R: CGGAAGTACGGCCTGAGAG |  |  |
|  | *Cyp19a1* | F: ATGGGGATTGGAAGTGCCTG | 109 | NM_007810.4 |
|  |  | R: ACATGCTTGAGGACTTGCTG |  |  |
| Pig | *GAPDH* | F: ACCCAGAAGACTGTGGATGG | 79 | NM_001206359.1 |
|  |  | R: AAGCAGGGATGATGTTCTGG |  |  |
|  | *GDF15* | F: TCAAGTCCGGATAGTCAC | 82 | NM_001174056.1 |
|  |  | R: AGTTAAGTTGACGCGAGG |  |  |
| Human | *GAPDH* | F: TCGGAGTCAACGGATTTGGT | 181 | NM_002046.7 |
|  |  | R: TTCCCGTTCTCAGCCTTGAC |  |  |
|  | *GDF15* | F: GCAAGAACTCAGGACGGTGA | 146 | NM_004864.4 |
|  |  | R: TGGAGTCTTCGGAGTGCAAC |  |  |
| ChIP-PCR  (mouse) | *Igf2* | F: GTCTGTGCAGAAAGGGCTG | 215 | NA |
|  |  | R: TCTGAGTGGCTCCTCCCAGT |  |  |
