# Supplementary material for: Deficiency of placental GDF15 causes fetal growth restriction by inhibiting fetal hepatic STAT3-IGF2 axis
Source: PLoS Biol. 2026 Oct 14;24(10):e3004061. doi: 10.1371/journal.pbio.3004061 (PMC13686457; doi:10.1371/journal.pbio.3004061)
Supplement: Supplementary file 4 [file pbio.3004061.s015.docx]

**S3 Table. Primary antibodies used in this study.**

| **Antibody** | **Source** | **Dilution (WB/IF/IHC)** | **Cat. No** |
| --- | --- | --- | --- |
| Anti-IGF2 | Abcam | 1:1000/1:200/no | ab308214 |
| Anti-p-STAT3 (Tyr705) | Cell Signaling Technology | 1:1000/no/no | 9145 |
| Anti-STAT3 | Proteintech | 1:1000/no/no | 10253-2-AP |
| Anti-GAPDH | Proteintech | 1:5000/no/no | 10494-1-AP |
| Anti-GDF15 | Santa Cruz | no/1:200/1:100 | sc-515675 |
| Anti-CYP19A1 | Proteintech | 1:2000/no/no | 16554-1-AP |
| Anti-Rabbit lgG | Abbkine | 1:5000/no/no | A21020 |
| Anti- Mouse lgG | Abbkine | no/no/1:500 | A21010 |
| Anti-Rabbit fluorescent antibody | Proteintech | no/1:200/no | RGAR002 |
| Anti-Mouse fluorescent antibody | Proteintech | no/1:200/no | RGAM002 |
