# Supplementary material for: Deficiency of placental GDF15 causes fetal growth restriction by inhibiting fetal hepatic STAT3-IGF2 axis
Source: PLoS Biol. 2026 Oct 14;24(10):e3004061. doi: 10.1371/journal.pbio.3004061 (PMC13686457; doi:10.1371/journal.pbio.3004061)
Supplement: Supplementary file 5 [file pbio.3004061.s016.docx]

**S4 Table. Linear mixed-effects model (LMM) analyses of fetal growth outcomes.**

| **Figure** | **Phenotype** | **Fixed effect** | ***P*** |
| --- | --- | --- | --- |
| Fig 1A | Fetal weight (g) | Group | <0.001 |
|  |  | Sex | 0.476 |
|  |  | Group x Sex | 0.983 |
| Fig 2F | Fetal weight (g) | Group | <0.001 |
|  |  | Sex | 0.697 |
|  |  | Group x Sex | 0.540 |
| Fig 2G | Crown-rump length (mm) | Group | 0.010 |
|  |  | Sex | 0.616 |
|  |  | Group x Sex | 0.818 |
| Fig 2J | Birth weight (g) | Group | <0.001 |
|  |  | Sex | 0.418 |
|  |  | Group x Sex | 0.999 |
| Fig 3I | Fetal weight (g) | Group | <0.001 |
|  |  | Sex | 0.535 |
|  |  | Group x Sex | 0.904 |
| Fig 3J | Crown-rump length (mm) | Group | <0.001 |
|  |  | Sex | 0.444 |
|  |  | Group x Sex | 0.777 |
| Fig 4G | Fetal weight (g) | Group | <0.001 |
|  |  | Sex | 0.280 |
|  |  | Group x Sex | 0.853 |
| Fig 4H | Crown-rump length (mm) | Group | <0.001 |
|  |  | Sex | 0.387 |
|  |  | Group x Sex | 0.991 |
| S1A Fig | Crown-rump length (mm) | Group | <0.001 |
|  |  | Sex | 0.489 |
|  |  | Group x Sex | 0.733 |
| S2F Fig | Fetal weight (g) | Group | 0.381 |
|  |  | Sex | 0.862 |
|  |  | Group x Sex | 0.491 |
| S2G Fig | Crown-rump length (mm) | Group | 0.186 |
|  |  | Sex | 0.429 |
|  |  | Group x Sex | 0.784 |
